# Language Enhanced Model for Eye (LEME): An Open-Source Ophthalmology-Specific Large Language Model

**Published:** 2024-10-01

**Authors:** Aidan Gilson, Xuguang Ai, Qianqian Xie, Sahana Srinivasan, Krithi Pushpanathan, Maxwell B. Singer, Jimin Huang, Hyunjae Kim, Erping Long, Peixing Wan, Luciano V. Del Priore, Lucila Ohno-Machado, Hua Xu, Dianbo Liu, Ron A. Adelman, Yih-Chung Tham, Qingyu Chen

**Affiliations:** 1. Department of Ophthalmology, Massachusetts Eye and Ear, Harvard Medical School, Boston, Massachusetts; 2. Department of Biomedical Informatics and Data Science, Yale School of Medicine, Yale University, New Haven, USA; 3. Singapore Eye Research Institute, Singapore National Eye Centre, Singapore; 4. Centre for Innovation and Precision Eye Health, Department of Ophthalmology, Yong Loo Lin School of Medicine, National University of Singapore, Singapore; 5. Department of Ophthalmology and Visual Science, Yale School of Medicine, Yale University, New Haven, USA; 6. Department of Computer Science, Korea University, 145 Anam-ro, Seongbuk-gu, Seoul, 02841, Republic of Korea; 7. Division of Cancer Epidemiology and Genetics, National Cancer Institute, National Institutes of Health, Bethesda, MD, USA; 8. Center for Cancer Research, National Cancer Institute, National Institutes of Health, Bethesda, MD, USA; 9. Ophthalmology and Visual Science Academic Clinical Program, Duke-NUS Medical School, Singapore, Singapore.

## Abstract

Large Language Models (LLMs) are poised to revolutionize healthcare. Ophthalmology-specific LLMs remain scarce and underexplored. We introduced an open-source, specialized LLM for ophthalmology, termed Language Enhanced Model for Eye (LEME). LEME was initially pre-trained on the Llama2 70B framework and further fine-tuned with a corpus of ~127,000 non-copyrighted training instances curated from ophthalmology-specific case reports, abstracts, and open-source study materials. We benchmarked LEME against eight other LLMs, namely, GPT-3.5, GPT-4, three Llama2 models (7B, 13B, 70B), PMC-LLAMA 13B, Meditron 70B, and EYE-Llama (another ophthalmology-specific LLM). Evaluations included four internal validation tasks: abstract completion, fill-in-the-blank, multiple-choice questions (MCQ), and short-answer QA. External validation tasks encompassed long-form QA, MCQ, patient EHR summarization, and clinical QA. Evaluation metrics included Rouge-L scores, accuracy, and expert evaluation of correctness, completeness, and readability.

In internal validations, LEME consistently outperformed its counterparts, achieving Rouge-L scores of 0.20 ± 0.03 in abstract completion (all p<0.05), 0.82 ± 0.04 in fill-in-the-blank (all p<0.0001), and 0.22 ± 0.05 in short-answer QA (all p<0.0001, except versus GPT-4). In external validations, LEME excelled in long-form QA with a Rouge-L of 0.19 ± 0.01 (all p<0.0001), ranked second in MCQ accuracy (0.68 ± 0.09; all p<0.0001), and scored highest in EHR summarization and clinical QA (ranging from 4.24 to 4.83 out of 5 for correctness, completeness, and readability).

LEME’s emphasis on robust fine-tuning and the use of non-copyrighted data represents a breakthrough in open-source ophthalmology-specific LLMs, offering the potential to revolutionize execution of clinical tasks while democratizing research collaboration.

## 1. Introduction

Large Language Models (LLMs) have rapidly advanced at the intersection of Generative Artificial Intelligence (AI) and Natural Language Processing (NLP).^1–3^ These models, characterized by billions of parameters, are pretrained on vast amounts of text data using self-supervised techniques^4–6^. Consequently, these models possess in-context learning capability,^7,8^ where they can interpret and generate text in a human-like manner, even with minimal prompts and on new tasks (i.e., zero or few-shot learning).^9–11^

To date, LLMs have demonstrated exceptional performances in general-domain tasks such as reading, comprehension, and question answering (QA)^4,12^.

In medicine, LLMs offer opportunities to enhance patient education, interaction, clinical documentation, and personalized medicine.^2,13–16^ However, most general-domain LLMs (e.g., ChatGPT, GEMINI) may lack access to most up-to-date medical knowledge, clinical evidence, and guidelines, resulting in inaccurate or even hallucinated responses.^17,18^

To address this issue, several medical-specific foundation models, such as PMC-LLAMA^19^, Meditron^20^, and Me-LLAMA^21^, have been developed. These medical foundation language models underwent two-stage training: a continuous pre-training on unsupervised domain-specific corpora, followed by instruction-tuning phase which fine-tuned the models with instructions for targeted inputs and outputs. For instance, PMC-LLAMA, one of the first medical LLMs, utilized Llama as the backbone and underwent continuous pretraining on 4.8 million biomedical literature articles from PubMed Central (PMC) and 30,000 medical books. It was subsequently instruction-tuned on conversations and curated QA datasets.^19,22^ However, results demonstrate that continuous pretraining is extremely resource-intensive (e.g., requiring hundreds of graphics processing units (GPUs), with minimal yield (e.g., an average increase of 2% of accuracy score in medical QA).^20^

In contrast, instruction-tuning directly from general-domain LLMs is potentially more effective for both resource efficiency and performance.^23^ For instance, eight A100 GPUs are typically sufficient to instruction-tune a Llama 70B.^21^ Recent studies also demonstrated that instruction-tuned LLMs possess robust zero-shot capabilities.^24,25^ Other medical domains such as pathology and radiology, have similarly adopted this approach to customize specialty-specific LLM.^26–29^

To that end, ophthalmology-specific LLMs remain scarce and underdeveloped. To date, there are three ophthalmology-specific LLMs (Supplementary Material S1)^30–32^. However, these three LLMs have several limitations. First, their past evaluations mainly focused on knowledge-based QA, which may not adequately assist patients (e.g., answering complex queries) or clinicians (e.g., summarizing patient cases) in clinical scenarios. Second, their evaluations were mostly limited to the same source as training data, lacking independent validations on new tasks or datasets.^33^ Therefore, their reliability and generalizability to other downstream ophthalmological applications remain uncertain. Third, these three LLMs were primarily fine-tuned on private or copyrighted data, making them less suitable for release as open-source. Additionally, these LLMs were mostly built on the smaller backbones (e.g., Llama2 7B) and had constrained instruction-following and reasoning capabilities compared to larger models.^4,5^

To address these current gaps, we developed a new ophthalmology-specific, open-source LLM, termed LEME (Language Enhanced Model for Eye). Leveraging the expansive Llama2 70B pre-trained framework, LEME was fine-tuned on a vast public dataset comprising of approximately 127,000 training instances from 19 designated tasks in ophthalmology. Hence, LEME is the most extensive ophthalmology-specific LLM to date. In this study, we comprehensively evaluated LEME against eight other LLMs, including general domain (closed- or open-source), medical-specific, and ophthalmology-specific models.

## 2. Methods

**Figure 1.**
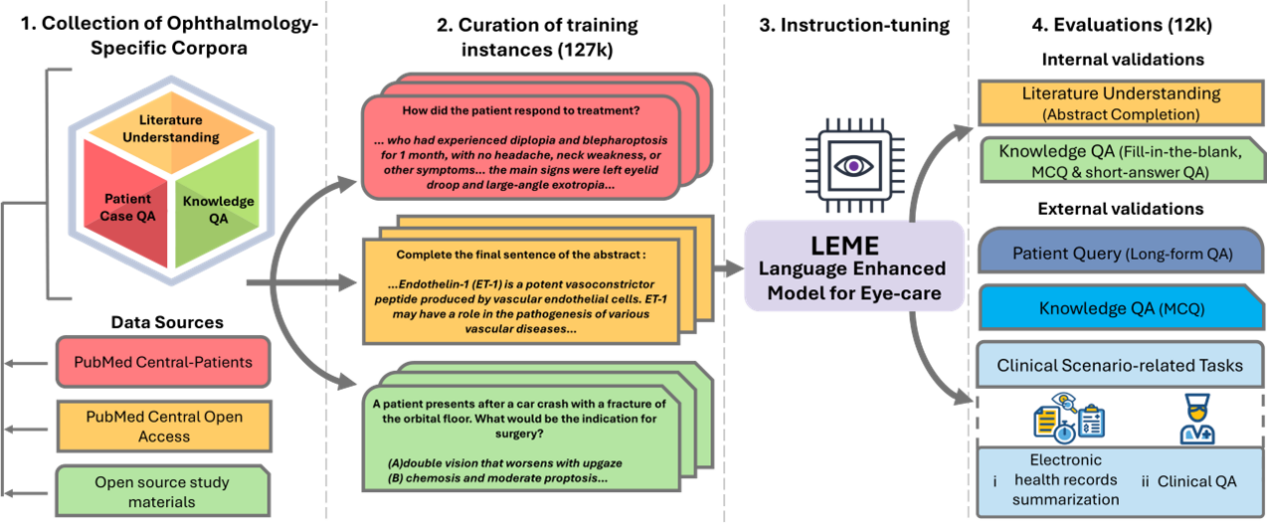
Overview of LEME’s Development and Evaluations

The development of LEME involved several key steps, beginning with the preparation of ophthalmology-specific corpora. We then curated instructions from these corpora to create a robust dataset of 127,000 training instances. Leveraging on this vast pool of training instances, LEME underwent instruction-tuning. Finally, we conducted comprehensive evaluations of LEME, including internal and external validations (including zero-shot learning tasks). Notably, we also assessed and compared LEME’s performance with eight other existing LLMs. Figure 1 provides an overview of the development and evaluation of LEME.

### 2.1 Preparation of Ophthalmology-Specific Corpora

We constructed three types of ophthalmology-specific corpora: patient case QA (question-answering), literature understanding, and knowledge QA. These corpora were collected from three primary sources: open-sourced patient case reports, relevant scientific literature, and study materials.

#### Ophthalmologic Patient Case Reports

From PMC-Patients, a data repository for patient case reports from PubMed Central (Open Access),^6,34^ we further collected 4,688 full-text case reports detailing various ophthalmologic conditions. To ensure these cases were indeed ophthalmology-relevant, we identified and cross-referenced the reports published in the ophthalmology-related journals (part of PubMed Central, see below).

#### Ophthalmology Journal Abstracts

From the PubMed Central (Open Access), we gathered 103,473 ophthalmology-related abstracts encompassing publications from fourteen leading ophthalmology journals: including Acta Ophthalmologica (2,331), American Journal of Ophthalmology (13,500), Asia-Pacific Journal of Ophthalmology (739), British Journal of Ophthalmology (11,288), Canadian Journal of Ophthalmology (2,881), Eye (6,910), Graefe’s Archive for Clinical and Experimental Ophthalmology (8,147), Investigative Ophthalmology and Visual Science (22,749), JAMA Ophthalmology (1,529), Journal of Cataract and Refractive Surgery (9,100), Ophthalmology (12,029 publications), Ophthalmology Glaucoma (341), Retina (6,055), Survey of Ophthalmology (2,408).

#### Ophthalmology Study Materials

We manually reviewed and amassed publicly available ophthalmology questions. These questions were posted by the community, including medical students, residents, and attending physicians. The users provided consent to allow these questions be made publicly available and ensured that the content did not violate copyright policy. To ensure a high-quality collection, we meticulously reviewed and selected only the relevant questions. Ultimately, we included 27,553 questions as study materials.

### 2.2 Curate Instructions from Ophthalmology-Specific Corpora

From these gathered corpora, we further curated 126,921 instructions across the three categories of patient case QA, literature understanding, and knowledge QA, encompassing 19 distinct tasks related to ophthalmology (Table 1). Each instruction contained related descriptions of the task, corresponding inputs, and outputs. This approach was consistent with existing LLM development studies.^35–37^ The detailed instruction format per task is summarized in Supplementary Material S2A, 2B and 2C.

#### Patient Case QA (encompassing 15 tasks)

We curated a comprehensive set of 15-task questions, encompassing diverse clinical perspectives including differential diagnosis and management (details in Supplementary Material S2A). 600 patient case reports were randomly selected from 4,688 patient cases to generate QA pairs. These questions were designed to mirror scenarios commonly encountered during patient queries or consultations. For each question, a corresponding answer was curated.

To efficiently handle the large number of answers for manual curation, we leveraged on the capabilities of both GPT-3.5 and GPT-4 to generate answers, treating them as “weak labels” (i.e. using automatically generated labels to fine-tune LLM in a scalable manner). Based on preliminary evaluations, we did not observe significant differences in the answers between GPT-3.5 and GPT-4. Considering the cost involved, GPT-3.5 was eventually chosen to generate the final QA pairs. Using this approach, we eventually generated 9,000 QA pairs in total.

#### Literature Understanding (1 task)

The abstract completion task was formulated by presenting the model with incomplete abstracts. Specifically, from abstracts extracted from the ophthalmology-related journals, we intentionally omitted the final sentence. We then prompted the model with an ‘instruction’ to ‘complete the abstract’. This task was applied to all 103,473 abstracts collected.

#### Knowledge QA (3 tasks)

Using the study questions derived from the open-source study materials in AnkiHub (described above), we further repurposed them into three question styles: fill-in-the-blank, multiple-choice questions (MCQ), and short-answer QA. We randomly sampled, manually reviewed the quality of the QA pairs, eventually arriving at 27,553 QA pairs in total.

### 2.3. Instruction-tuning

Our model, LEME first leveraged the Llama2 70B framework as its backbone (utilizing its open-source pretrained parameter weights). We then fine-tuned LEME based on the ~127,000 curated instructions described above.

#### Backbone Model

LLaMA2 70B was used as the backbone model. LLaMA2 is a family of pre-trained LLMs developed by Meta AI. LLaMA2 has several sizes of model, up to 70 billion parameters.^4^ The architecture of LLaMA2 builds upon the standard Transformer model with several key modifications to positional encodings and attention mechanisms, further optimizing its efficiency and capability.^38^ Furthermore, LLaMA2 is pre-trained on a larger and more diverse dataset compared to its predecessor LLaMA, enabling it to better understand and generate human-like text.^5,39,40^ The detailed description of LLaMA2 is provided in the Supplementary Material’s Appendix section.

#### Training function

The objective function used for instruction tuning used was: $L(\theta )=\sum \frac{1}{\left|r\right|}log(r|i,\theta )$, where i represents the input instructions, r denotes the model’s responses, and θ signifies the parameter set of the model. This function was designed to optimize the model parameters by maximizing the likelihood of the model predicting the correct responses to given instructions.

#### Training/ Fine-tuning

The training was performed by fine-tuning the model on the curated instructions (as detailed above). Specifically, the curated instructions were randomly split, allocating 90% for training and 10% for internal validation. (Table 1) The training was conducted on eight H100 80G GPUs, spanning three epochs with predefined hyperparameters including a learning rate of 1e−5, a weight decay factor of 0.00001, and a warmup ratio of 0.01. Low-Rank Adaptation (LoRA) parameter-efficient tuning technique were employed to improve training efficiency.^6,41^ Further descriptions are provided in the Supplementary Material’s Appendix section.

### 2.4 Evaluation

We benchmarked LEME against eight other LLMs: GPT-3.5, GPT-4, Llama2 frameworks (7B, 13B, 70B), PMC-LLAMA 13B, Meditron 70B, and EYE-Llama. Evaluations included internal validation tasks (abstract completion, fill-in-the-blank, MCQ, and short-answer QA) and external validation tasks (long-form QA, MCQ, patient EHR summarization, and clinical QA).

#### Benchmarking models

Eight LLMs were selected for comparison: GPT-3.5 (gpt-3.5-turbo-0613) and GPT-4 (gpt-4–0613) as closed-source representatives; Llama2 (7B, 13B, 70B) as open-source general-domain representatives; PMC-LLAMA 13B and Meditron 70B as medical-specific representatives; and EYE-Llama as an ophthalmology-specific LLM. In this main benchmarking evaluation, EYE-Llama was evaluated in its pretrained form (EYE-Llama_p version, i.e. without being fine-tuned on specific downstream tasks.^7^

All LLMs were evaluated using standardized instruction templates (Supplementary Material S2A, S2B, and S2C) with the ‘temperature’ set to 0 to minimize response variance. To address response format variability across the evaluated LLMs, targeted predictions were extracted from raw responses using automated text processing and were manually reviewed.

#### Internal Validations

During internal validation, a random subset comprising 10% of the curated instructions was selected for testing (Table 1). It encompassed four distinct tasks: abstract completion, fill-in-the-blank, MCQ, and short-answer QA (curation details were as described above).

#### External Validations

These external evaluations benchmarked LLMs in a zero-shot setting (i.e. without prior training). Here, we assessed the models based on long-form QA, MCQ and clinical scenario-related tasks (patient EHR summarization and clinical QA) (Table 1).

#### Long-form QA Task

We evaluated 76 long-form QAs sourced from the Ask an Ophthalmologist forum from American Academy of Ophthalmology’s (AAO), containing patient health inquiries answered by certified ophthalmologists.^42^ This task represents zero-shot testing as it was absent from LEME’s training data. Questions were randomly sampled from subtopics including Retina, Glaucoma, Cataracts, Dry Eye, and Uveitis to ensure diverse coverage.

#### MCQ Task

We used 260 MCQs from the AAO’s Basic and Clinical Science Course Complete Set (BCSC), which covers a comprehensive range of ophthalmic knowledge.

#### Clinical Scenario Tasks

For clinical scenario-related tasks (patient EHR summarization and clinical QA), we evaluated Llama2 70B, EYE-Llama, and LEME. This evaluation involved data from the EHR database of the Eye Center at Yale School of Medicine and was performed on a HIPAA-compliant server with IRB approval (protocol number 2000037687). As these tasks required human expert evaluation and were labor intensive, we only selected Llama2 70B (the base model of LEME) and EYE-Llama (being another ophthalmology-specific) models. Additionally, GPT-3.5 and GPT-4 were excluded due to confidentiality concerns as these tasks involved data from EHR database of a health institution.

These three models were assessed using 27 randomly sampled patient notes from the EHRs, comprising 9 cases each for retinal, glaucoma, and anterior segment clinical encounters. In the patient EHR summarization task, all three evaluated models were prompted to generate a one-liner summary following a structured format, including key elements such as patient age, gender, past medical history, chief complaint, and relevant history or concurrent symptoms. This evaluation focused on assessing the model’s proficiency in clinical summarization in structured format, emphasizing accurate case detail presentation while avoiding unnecessary information repetition. For clinical QA, the models were prompted to respond to four questions after receiving a patient’s EHR: “What was the work-up? What did the slit lamp exam demonstrate? What treatment was provided to the patient? What is the expected clinical course of the patient following the outlined treatment?”. These questions were designed to emulate typical clinician assessments during patient reviews.

Two ophthalmologists independently assessed the responses of the LLMs based on the evaluation metrics of correctness (is the response correct?), completeness (does the response capture the key information?), and readability (is the response easy to read?). These metrics used a 5-point scale (Supplementary Material S3). To mitigate bias, the order of the responses was randomized, and the evaluators were blinded to the LLMs. Final scores were determined as the average of both evaluators’ ratings.

#### Additional head-to-head comparisons with fine-tuned versions of EYE-Llama

The purpose of this additional evaluation was to perform a more leveled head-to-head comparisons with EYE-Llama’s fine-tuned versions, namely, EYE-Llama_qa and EYE-Llama_gqa.^1^ EYE-Llama_qa was fined tuned on 10 sets of open-sourced medical related QAs, consisting of 20k QA pairs, while EYE-Llama_gqa was further fine-tuned with an additional 15,000 QAs synthetically generated by ChatGPT-3.5. For direct and fair comparisons, we evaluated EYE-Llama_qa and EYE-Llama_gqa in both long-form QA and MCQ tasks under external validation in our study. Additionally, we evaluated LEME using the MedMCQA subset (780 instance), which was used as a testing set in the original EYE-Llama paper.^30^

#### Evaluation Metrics and Statistical Analysis

To evaluate the performance of LEME and other benchmarked LLMs, we utilized a variety of primary and secondary evaluation metrics across different tasks in both internal and external validations. These evaluation metrics are commonly adopted for quantifying the performance of LLMs, and are summarized in Table 2.^8^

For abstract completion, fill-in-the-blank, short-answer QA, long-form QA tasks, the primary evaluation metric was Rouge-L (higher score indicates better alignment with the reference text), while BERT Score and BART Score served as secondary metrics. A higher BERT score indicates closer semantic match to the reference text. BART score was expressed as the log-likelihood of probabilities, less negative value indicates a closer match to the reference text^43,44^. For MCQs, accuracy was the primary metric, complemented by Macro-F1 score as secondary metric. Additionally, patient EHR summarization and clinical QA tasks were assessed based on correctness, completeness, and readability, evaluated on a 5-point scale by two independent ophthalmologists. Final scores were determined as the average of both evaluators’ ratings.

To quantify statistical significance for all internal validations, as well as long-form QA and MCQ tasks in the external validations, we performed bootstrapping with a sampling size of 30 and repeated the process 100 times. We then performed a two-tailed Wilcoxon rank-sum test with a 95% confidence interval. We also applied the Bonferroni correction to account for multiple test comparisons.

## 3. Results

### 3.1 Internal Validations

**Figure 2.**
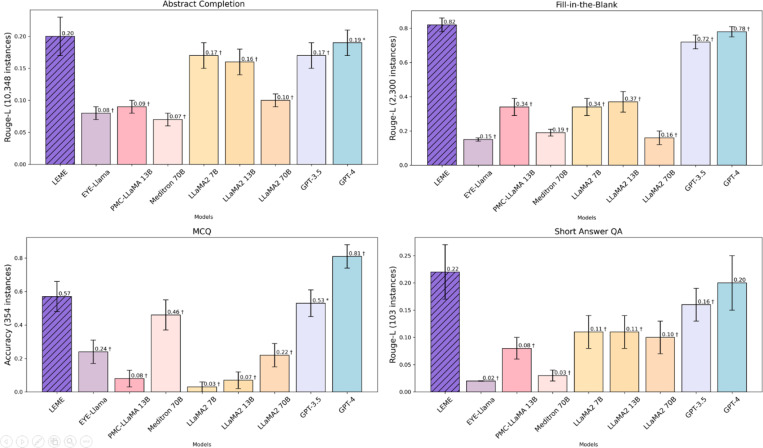
Internal Validation Results for LEME and compared to Eight Benchmark Models * denotes p-value < 0.05 and † denotes p-value < 0.0001 (after Bonferroni correction) for comparisons against LEME’s performance.

Figure 2 and Table 3 illustrate LEME’s performance in internal validations, as well as its benchmarking against eight other LLMs. LEME achieved Rouge-L scores of 0.20 ± 0.03 in abstract completion, 0.82 ± 0.04 in fill-in-the-blank, and 0.22 ± 0.05 in short-answer QA. For the MCQ task, LEME attained an accuracy score of 0.57 ± 0.09. Across the three tasks of abstract completion, fill-in-the-blank, and short-answer QA, LEME outperformed the eight other LLMs (P ≤ 0.0142 for all comparisons, except GPT-4 for short-answer QA). Based on BERT and BART score, LEME similarly achieved optimal performance, especially for fill-in-the-blank and short-answer QA tasks (Supplementary Material S4A).

Overall, the superior performance of LEME over other LLMs suggests that the instruction-tuning performed was highly effective for enhancing LEME’s performance. However, it is important to note that these findings are based on internal validations, where LEME was tested on dataset similar to its instruction-tuning set. To better assess the model’s generalizability, further consideration should be given to the results of external validations (see section below).

### 3.2 External Validations

**Figure 3.**
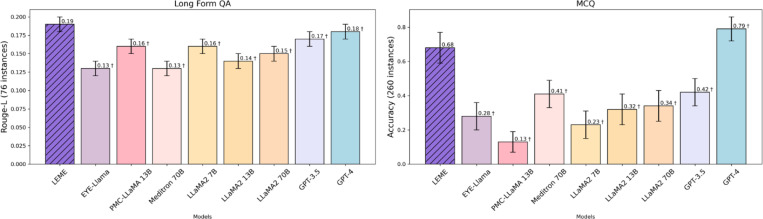
External Validation Results for LEME and compared to Eight Benchmark Models † denotes p-value < 0.0001 (after Bonferroni correction) for comparisons against LEME’s performance.

In external validation tasks of addressing patient queries (long-form QA) and knowledge QA (MCQs), Figure 3 and Table 3 illustrate the primary performance metrics. In long-form QA task, LEME outperformed other models (all p<0.0001; Table 3). Specifically, LEME achieved a Rouge-L score of 0.19 ± 0.01, compared to 0.13 ± 0.01 for EYE_Llama, 0.16 ± 0.01 for PMC-LLAMA, 0.13 ± 0.01 for Meditron, 0.16 ± 0.01 LLaMA2 7B, 0.14 ± 0.01 for LLaMA2 13B, 0.15 ± 0.01 for LLaMA2 70B, 0.17 ± 0.01 for GPT-3.5, and 0.18 ± 0.01 for GPT-4. It was also observed that PMC-LLAMA and EYE-Llama provided inaccurate and hallucinated responses (Supplementary Material S5). In the MCQ task, LEME (accuracy score of 0.68 ± 0.09) performed better than other models (all P<0.0001), except for GPT-4, which achieved a higher score of 0.79 ± 0.07 (Table 3). Furthermore, in the long-form QA task, LEME’s BERT score (0.72) was consistently higher than those of other LLMs and was similar to GPT-4 (0.72). In the MCQ task, LEME’s Macro-F1 score (0.68) was also higher than those of other models, except for GPT-4 (0.77) (Supplementary Material 4B).

**Figure 4.**
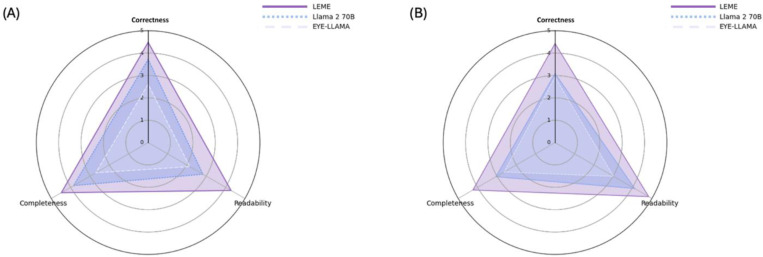
Radar Charts Summarizing the Ratings by Two Ophthalmologists for LEME, LLaMA 2 70B, and EYE-Llama on Correctness, Completeness, and Readability for (A) Patient EHR Summarization and (B) Clinical QA Across 27 Patient EHRs

Additionally, we evaluated LEME’s performance against its non-fine-tuned backbone model, Llama2 70B, and an ophthalmology-specific LLM, EYE-Llama, on two clinical scenario-related tasks: patient EHR summarization and Clinical QA. Overall, LEME consistently outperformed Llama2 70B and EYE-Llama across all evaluated metrics and tasks (Table 4, Figure 4, and Supplementary Material S6). For patient EHR summarization, in terms of correctness, LEME achieved the highest mean score of 4.48, followed by Llama2 70B (3.72), and EYE-Llama (2.61). For completeness, LEME achieved a score of 4.48, followed by Llama2 70B (3.85), and EYE-Llama (2.70). In readability, LEME achieved a higher score of 4.28, compared to 2.83 for Llama2 70B and 2.15 for EYE-Llama. In the Clinical QA task, LEME also performed better, achieving higher correctness score of 4.43, compared to Llama2 70B (3.09) and EYE-Llama (2.93). For completeness, LEME achieved a score of 4.24, followed by Llama2 70B (3.06), and EYE-Llama (2.63). For readability in clinical QA task, LEME achieved a higher score of 4.83, compared to Llama2 70B (4.11) and EYE-Llama (3.02). Examples of the patient EHR notes, the standardized questions, and the corresponding responses generated by the three models are presented in Supplementary Material S7.

### 3.3 Head-to-head comparisons with another ophthalmology-specific LLM in zero-shot conditions

We additionally performed head-to-head comparisons between LEME and the fine-tuned versions of EYE-Llama (i.e. EYE-Llama_qa and EYE-Llama_gqa). LEME outperformed both fine-tuned versions in the external validation tasks of long-form QA and MCQ (see Supplementary Material S8). In long-form QA, LEME achieved a higher Rouge-L score of 0.19 ± 0.01 compared to EYE-Llama_qa’s score of 0.18 ± 0.01 and EYE-Llama_gqa’s score of 0.17 ± 0.01. In the MCQ task, LEME achieved an accuracy of 0.68 ± 0.09, higher than EYE-Llama_qa (0.21 ± 0.01) and EYE-Llama_gqa (0.24 ± 0.08).

Furthermore, when benchmarked on the 780 subset of MEDMCQA dataset (which was used as the evaluation set in the original EYE-Llama study), LEME achieved an accuracy of 0.47, outperforming both EYE-Llama_qa (0.35) and EYE-Llama_gqa (0.39) (Supplementary Material S9). In this instance, it is also noteworthy that EYE-Llama_qa and EYE-Llama_gqa were partially fine-tuned on the MEDMCQA training set, whereas LEME was tested in a zero-shot setting.

## 4. Discussion

This study presents the development and validation of LEME, the most extensive ophthalmology-specific LLM to date. Leveraged on the expansive Llama2 70B pre-trained framework, we fine-tuned the model with a substantial dataset comprising approximately 127,000 customized instructions derived from ophthalmology case reports, abstracts, and open-source study materials. When compared to eight existing LLMs, LEME demonstrates superior performance across a variety of tasks. Notably, this study is pioneering in evaluating ophthalmology-specific LLMs on clinical scenario-related tasks involving EHR data, an aspect often lacking in prior studies. LEME signifies a significant breakthrough in the field, with the potential to transform patient query services, clinical workflows, and the delivery of eye care services. Furthermore, its open-source nature promotes innovation and enables continuous improvement by the broader community, facilitating the adaptation and enhancement of other ophthalmology-specific models. Our approach, which emphasizes robust fine-tuning and openness, may serve as a learning model for other medical domains.

In internal validations, LEME outperformed the benchmarking LLMs in all tasks except MCQs, where it ranked second to GPT-4 (Figure 2 and Table 3). This internal validation is necessary to quantify the effectiveness of instruction fine-tuning, demonstrating the improvement of LEME from its pretrained backbone LLaMA2 70B and other LLMs. The results also demonstrated that LEME can learn multiple tasks simultaneously, consistent with the results reported by other studies on instruction fine-tuning of LLMs in the medical domain.^35,45^ Nevertheless, given that LEME was tested on data similar to its instruction-tuning set in these internal validations, external validation is warranted and was performed as well.

In external validations (zero-shot conditions) all involved LLMs were evaluated under the same setting. LEME demonstrated superior zero-shot performance in new tasks (i.e. data was previously not trained on within external validations, such as long-form QA and clinical case scenarios). For instance, LEME outperformed all eight benchmarking models on long-form QA (Figure 3 and Table 3). LEME surpassed both LLaMA2 70B, its backbone model, and EYE-Llama, the only other publicly available ophthalmology-specific LLM, in clinical case scenarios like EHR summarization and clinical QA (Figure 4). LEME achieved the highest correctness, completeness, and readability across these tasks (Table 4). These findings, especially on the general open-sourced foundation models (i.e. Llama2 variants), collectively suggest that continuous pre-training might not be effective for domain-specific LLMs due to significant computational costs without proportional improvement in performance. Instead, instruction tuning on diverse tasks appears more effective and with reasonable computational costs. Nonetheless, fully capturing domain-specific knowledge during this process remains a challenge, and more efficient curation of training instances across corpora ought to be explored in future work. Interestingly, we observed that closed-source LLM baselines (GPT-3.5 and GPT-4) consistently had better performance than other baseline models including medical LLMs (PMC-LLAMA and Meditron) in both internal and external validations. The previous literature also reported similar observations.^9,10,46^ This indicates that general-domain LLMs do not necessarily underperform compared to medical LLMs.^13,47,48^ For instance, PMC-LLAMA struggled to follow instructions, while Meditron provided hallucinated content in its responses (Supplementary Material S5, see sections Abstract completion, Fill-in-the-blank, Short-answer QA of Internal Validation and MCQ of External Evaluation).In contrast, LEME outperformed GPT-3.5 in all the tasks evaluated and surpassed GPT-4 in all most tasks except MCQ. Prior research has shown similar results with GPT-4 excelling in MCQ tasks,^30,49,50^ possibly due to its advanced reasoning capabilities.^51,52^ This suggests a potential area for enhancement in LEME. Nevertheless, it is important to note that GPT-3.5 and GPT-4 are closed-source LLMs with inaccessible training data, potentially limiting pure external validation. It cannot be ascertained if the datasets used in external validation were ever used by GPT-3.5 or GPT-4 for training or fine-tuning. For example, it is unclear whether GPT-4’s superior performance on MCQs is due to advanced reasoning capabilities or because it was trained on a large amount of data, including similar ophthalmological sources.^49^. In contrast, LEME utilized non-copyrighted data with full disclosure of its fine-tuning dataset, making it better suited as a source for direct benchmarking with other LLMs by the research community.

When comparing LEME with another ophthalmology-specific LLM, EYE-Llama, we observed that LEME’s performance was better, especially in long-form QA and external MCQ task (Table 3). We also observed EYE-Llama exhibited hallucinations in its responses. For example, when queried about cataract surgery and Laser-Assisted in Situ Keratomileusis (LASIK) surgery, EYE-Llama provided incorrect information and referenced studies related to training and testing of AI models which were irrelevant. (Supplementary Material S5). Further to this, we also performed additional head-to-head comparisons with EYE Llama’s fine-tuned version (EYE-Llama_qa and EYE-Llama_gqa). Across all tasks in external validations, including using EYE-Llama’s original study’s validation dataset MEDMCQA, LEME consistently outperformed EYE-Llama’s fine-tuned versions (Supplementary Material S8 and S9). Additionally, LEME achieved up to 12% higher accuracy than EYE-Llama_qa and EYE-Llama_gqa on the MEDMCQA subset (Supplementary Material S9). Notably, on the MEDMCQA validation, LEME can be considered as being tested in a zero-shot setting, whereas both EYE-Llama_qa and EYE-Llama_gqa were not, as both were fine-tuned using the MEDMCQA subset. Despite this, EYE-Llama’s performance, both in its pre-trained form and its fine-tuned variants, remained inferior to LEME’s.

Currently, medical domain-LLMs are still mostly confined to knowledge testing (e.g. fill-in-the blank etc.).^15,17,53,54^ These are challenges common to medical domain-LLMs. To truly transform clinical practice, the goal is to leverage LLMs to alleviate the workload of healthcare professionals, for instance, by assisting in diagnosis and drafting clinical notes.^15^ Achieving this broader application necessitates the incorporation of real-world clinical data, including patient notes from EHRs, for fine-tuning purposes. However, the availability of the necessary computational infrastructure for secure instruction-tuning using EHRs is currently still limited. Additionally, the absence of standardized benchmarking datasets in ophthalmology also poses a significant challenge for objective comparisons. Furthermore, LLMs are frequently evaluated using varying metrics, complicating reproducibility and hindering direct comparisons. Establishing tailored benchmark datasets and setting guidelines for standardized evaluation methods are essential to address these issues and advance the development and application of LLMs in ophthalmology.

Our study has several strengths. First, the uniqueness of this study lies in its comprehensive methodology for model development, fine-tuning and multifaceted validations. LEME leveraged on the expansive LLaMA2 70B framework and was trained on a vast ophthalmology-specific dataset of 127,000 training instances, making it the most comprehensive ophthalmology-specific LLM developed to date. Furthermore, rigorous assessments against eight other existing LLMs across various tasks in internal and external validations further highlight LEME’s robustness and zero-shot learning capabilities. Third, in our evaluations, we utilized a comprehensive range of metrics, including Rouge-L, BERT, BART, accuracy, and F1-score, ensuring thorough performance assessment. Lastly, by adopting an open-source approach, we made our model publicly available for benchmarking, and potentially streamlining the workload for others to fine-tune their subsequent model for additional ophthalmological tasks. This is particularly important given the rapid and evolving demands of the LLM field.

This study also has some limitations. First, while we evaluated eight LLMs of different types (generic, medical-domain, and ophthalmology-specific), it was not feasible to include all available LLMs in the field for comparison against LEME. Nevertheless, the selected LLMs are strong representatives in this field based on their relevance and usage. Second, although we assessed the model for patient EHR summarization and clinical QA tasks, our study did not encompass the full spectrum of potential downstream clinical applications where LLMs could streamline workflows. Hence, in the next phase of future work, we plan to conduct more comprehensive validations focusing on clinical use cases such as diagnostic assistance, treatment recommendation, and patient education. These evaluations will help us to better understand LEME’s strengths and weaknesses across various clinical contexts.

## 5. Conclusion

LEME’s emphasis on robust fine-tuning and the use of non-copyrighted data represents a breakthrough in open-source ophthalmology-specific LLMs, offering the potential to revolutionize execution of clinical tasks. With its open-source availability, LEME fosters wider collaboration across the LLM research community and encourages continuous refinement and adaptation by others.

## Supplementary Material

Supplement 1

## Figures and Tables

**Table 1:** Data Distribution Across Training, Internal Validation and External Validation Sets

	TRAINING ONLY^†^	TRAINING & INTERNAL VALIDATION		EXTERNAL VALIDATION			
***DATASET CATEGORY***	Patient Case QA	Literature Understanding	Knowledge QA	Patient Query	Knowledge QA	Clinical Scenario-related Tasks	
***DATASET SOURCE***	Patient Case Reports from PubMed Central-Patients	Abstracts from PubMed Central Open Access	Open source, community-based Ophthalmology Questions/study materials	Ask An Ophthalmologist forum	BCSC*	Electronic Health Records (EHR) from Eye Center at Yale New Haven Hospital	
***TASK TYPE***	Diverse clinical perspectives including differential diagnosis and management	Abstract completion	Fill-in-the-blank, MCQ & Short-answer QA	Long-form QA	MCQ	Patient EHR Summarization	Clinical QA
***NUMBER OF TASKS***	15	1	3	1	1	1	4
***NUMBER OF INSTANCES***	9,000	103,473	27,553	76	260	27	108
***RATIO OF TRAINING TO VALIDATION DATA***	NA; not used for validation	9 : 1 (93,125:10,348)	9 : 1 (24796:2,757)	NA; not used for training			

† Training in this context refers to instruction-tuning;

* American Academy of Ophthalmology’s Basic and Clinical Science Course

**Table 2.** Evaluation Metrics for Internal and External Validations of LEME and Eight Benchmark LLMs

Evaluation Setting	Task	Primary Evaluation Metric	Secondary Evaluation Metrics
Internal validation	Abstract completion	Rouge-L	BERT Score, BART Score
	Fill-in-the-blank	Rouge-L	BERT Score, BART Score
	MCQ	Accuracy	Macro-F1
	Short-answer QA	Rouge-L	BERT Score, BART Score

External validation	Long-form QA	Rouge-L	BERT Score, BART Score
	MCQ	Accuracy	Macro-F1
	Patient EHR summarization	Correctness, Completeness, and Readability	-
	Clinical QA	Correctness, Completeness, and Readability	-

**Table 3.** Internal and External Validation Results for LEME compared to Eight Benchmark Models, Evaluated on Primary Metrics [Presented as Mean ± SD (95% CI)]

Task (Evaluation Metric)	Reference	Ophthalmology-specific	Medical-Specific		General Open-sourced			Close-sourced	
	LEME	EYE-Llama	PMC-LLAMA 13B	Meditron 70B	Llama2 7B	Llama2 13B	Llama2 70B	GPT-3.5	GPT-4
**Internal validation**									
Abstract completion (Rouge-L)	**0.20 ± 0.03 (0.15, 0.25)**	0.08 ± 0.01^†^ (0.06, 0.09)	0.09 ± 0.01^†^ (0.07, 0.12)	0.07 ± 0.01^†^ (0.06, 0.09)	0.17 ± 0.02^†^ (0.13, 0.20)	0.16 ± 0.02^†^ (0.12, 0.19)	0.10 ± 0.01^†^ (0.08, 0.13)	0.17 ± 0.02^†^ (0.14, 0.20)	0.19 ± 0.02* (0.16, 0.22)
Fill-in-the-blank (Rouge-L)	**0.82 ± 0.04 (0.76, 0.89)**	0.15±0.01^†^ (0.13, 0.18)	0.34 ± 0.05^†^ (0.26, 0.46)	0.19±0.02^†^ (0.14, 0.23)	0.34 ± 0.05^†^ (0.26, 0.44)	0.37 ± 0.06^†^ (0.27, 0.47)	0.16 ± 0.04^†^ (0.10, 0.23)	0.72 ± 0.04^†^ (0.64, 0.81)	0.78 ± 0.03^†^ (0.73, 0.85)
MCQ (Accuracy Score)	0.57 ± 0.09 (0.42, 0.73)	0.24 ± 0.07^†^ (0.12, 0.37)	0.08 ± 0.05^†^ (0.00, 0.17)	0.46 ± 0.09^†^ (0.28, 0.60)	0.03 ± 0.03^†^ (0.00, 0.10)	0.07 ± 0.05^†^ (0.00, 0.20)	0.22 ± 0.07^†^ (0.10, 0.37)	0.53 ± 0.08* (0.37, 0.67)	**0.81 ± 0.07**^†^ **(0.67, 0.93)**
Short-answer QA (Rouge-L)	**0.22 ± 0.05 (0.18, 0.30)**	0.02 ± 0.00^†^ (0.01, 0.03)	0.08 ± 0.02^†^ (0.04, 0.11)	0.03 ± 0.01^†^ (0.02, 0.04)	0.11 ± 0.03^†^ (0.06, 0.18)	0.11 ± 0.03^†^ (0.05, 0.17)	0.10 ± 0.03^†^ (0.06, 0.18)	0.16 ± 0.03^†^ (0.10, 0.23)	0.20 ± 0.05 (0.11, 0.32)
**External validation**									
Long-form QA (Rouge-L)	**0.19 ± 0.01 (0.17, 0.21)**	0.13 ± 0.01^†^ (0.11, 0.15)	0.16 ± 0.01^†^ (0.14, 0.18)	0.13 ± 0.01^†^ (0.11, 0.15)	0.16 ± 0.01^†^ (0.15, 0.18)	0.14 ± 0.01^†^ (0.12, 0.15)	0.15 ± 0.01^†^ (0.14, 0.16)	0.17 ± 0.01^†^ (0.16, 0.19)	0.18 ± 0.01^†^ (0.16, 0.19)
MCQ (Accuracy Score)	0.68 ± 0.09 (0.53, 0.87)	0.28 ± 0.08^†^ (0.13, 0.43)	0.13 ± 0.06^†^ (0.03, 0.27)	0.41 ± 0.08^†^ (0.25, 0.60)	0.23 ± 0.08^†^ (0.10, 0.38)	0.32 ± 0.09^†^ (0.17, 0.48)	0.34 ± 0.09^†^ (0.18, 0.52)	0.42 ± 0.08^†^ (0.27, 0.57)	**0.79 ± 0.07**^†^ **(0.67, 0.92)**

* Denotes p-value<0.05 and

† denotes p-value < 0.0001 (after Bonferroni correction) for comparisons against LEME’s performance.

**Table 4.** Head-to head performance of LEME against Llama2 70B and EYE-Llama on Patient EHR Summarization and Clinical QA Tasks, Based on Evaluations by Two Ophthalmologists

	LEME	Llama2 70B	EYE-Llama
**Patient EHR summarization**			

Correctness	**4.48**	3.72	2.61
Completeness	**4.48**	3.85	2.70
Readability	**4.28**	2.83	2.15

**Clinical QA**			

Correctness	**4.43**	3.09	2.93
Completeness	**4.24**	3.06	2.63
Readability	**4.83**	4.11	3.02

## Data Availability

LEME, related data, and codes are publicly available through the open-access GitHub repository at https://github.com/qingyu-qc/leme_eye_llm.

## References

[1] Howell MD, Corrado GS, DeSalvo KB. Three Epochs of Artificial Intelligence in Health Care. JAMA 2024; 331: 242–4. 38227029 10.1001/jama.2023.25057

[2] Betzler BK, Chen H, Cheng C-Y, Large language models and their impact in ophthalmology. The Lancet Digital Health 2023; 5: e917–24.38000875

[3] The Lancet Digital Health. Large language models: a new chapter in digital health. The Lancet Digital Health 2024; 6: e1. 38123249 10.1016/S2589-7500(23)00254-6

[4] Zhao WX, Zhou K, Li J, A survey of large language models. arXiv preprint arXiv:230318223 2023.

[5] Wang Y, Zhong W, Li L, Aligning large language models with human: A survey. arXiv preprint arXiv:230712966 2023.

[6] Zhang S, Dong L, Li X, Instruction tuning for large language models: A survey. arXiv preprint arXiv:230810792 2023.

[7] Lampinen AK, Dasgupta I, Chan SC, Can language models learn from explanations in context? arXiv preprint arXiv:220402329 2022.

[8] Lu S, Bigoulaeva I, Sachdeva R, Madabushi HT, Gurevych I. Are Emergent Abilities in Large Language Models just In-Context Learning? arXiv preprint arXiv:230901809 2023.

[9] Kojima T, Gu SS, Reid M, Matsuo Y, Iwasawa Y. Large Language Models are Zero-Shot Reasoners. ArXiv 2022; abs/2205.11916.

[10] Wang T, Roberts A, Hesslow D, What Language Model Architecture and Pretraining Objective Work Best for Zero-Shot Generalization? ArXiv 2022; abs/2204.05832.

[11] Chen S, Guevara M, Moningi S, The effect of using a large language model to respond to patient messages. The Lancet Digital Health 2024; 6: e379–81. 38664108 10.1016/S2589-7500(24)00060-8 PMC11829255

[12] Huang J, Chang KC-C. Towards reasoning in large language models: A survey. arXiv preprint arXiv:221210403 2022.

[13] Lee P, Bubeck S, Petro J. Benefits, Limits, and Risks of GPT-4 as an AI Chatbot for Medicine. New England Journal of Medicine 2023; 388: 1233–9. 36988602 10.1056/NEJMsr2214184

[14] Clusmann J, Kolbinger FR, Muti HS, The future landscape of large language models in medicine. Communications medicine 2023; 3: 141. 37816837 10.1038/s43856-023-00370-1 PMC10564921

[15] Shah NH, Entwistle D, Pfeffer MA. Creation and adoption of large language models in medicine. Jama 2023; 330: 866–9. 37548965 10.1001/jama.2023.14217

[16] Thirunavukarasu A, Ting D, Elangovan K, Gutierrez L, Tan T, Ting D. Large language models in medicine. Nat Med 2023; 29: 1930–40. 37460753 10.1038/s41591-023-02448-8

[17] Tian S, Jin Q, Yeganova L, Opportunities and challenges for ChatGPT and large language models in biomedicine and health. Briefings in Bioinformatics 2024; 25: bbad493. 10.1093/bib/bbad493 PMC10762511 38168838

[18] Umapathi LK, Pal A, Sankarasubbu M. Med-halt: Medical domain hallucination test for large language models. arXiv preprint arXiv:230715343 2023.

[19] Wu C, Lin W, Zhang X, Zhang Y, Xie W, Wang Y. PMC-LLaMA: toward building open-source language models for medicine. Journal of the American Medical Informatics Association 2024. DOI:10.1093/jamia/ocae045. PMC11639126 38613821

[20] Chen Z, Cano AH, Romanou A, Meditron-70b: Scaling medical pretraining for large language models. arXiv preprint arXiv:231116079 2023.

[21] Xie Q, Chen Q, Chen A, Me LLaMA: Foundation Large Language Models for Medical Applications. arXiv preprint arXiv:240212749 2024.

[22] Wu J, Liu X, Li M, Clinical Text Datasets for Medical Artificial Intelligence and Large Language Models — A Systematic Review. NEJM AI 2024; 1: AIra2400012.

[23] Vaid A, Landi I, Nadkarni G, Nabeel I. Using fine-tuned large language models to parse clinical notes in musculoskeletal pain disorders. The Lancet Digital Health 2023; 5: e855–8. 10.1016/S2589-7500(23)00202-9 39492289

[24] Li J, Pan K, Ge Z, Fine-tuning Multimodal LLMs to Follow Zero-shot Demonstrative Instructions. 2023.

[25] Nayak NV, Nan Y, Trost A, Bach SH. Learning to Generate Instruction Tuning Datasets for Zero-Shot Task Adaptation. ArXiv 2024; abs/2402.18334.

[26] Lu MY, Chen B, Williamson DFK, A Multimodal Generative AI Copilot for Human Pathology. Nature 2024; published online June 12. DOI:10.1038/s41586-024-07618-3. PMC11464372 38866050

[27] Blankemeier L, Cohen JP, Kumar A, Merlin: A Vision Language Foundation Model for 3D Computed Tomography. Research Square 2024.

[28] Lu MY, Chen B, Williamson DFK, A visual-language foundation model for computational pathology. Nature Medicine 2024; 30: 863–74. 10.1038/s41591-024-02856-4 PMC11384335 38504017

[29] Omar M, Ullanat V, Loda M, Marchionni L, Umeton R. ChatGPT for digital pathology research. The Lancet Digital Health 2024; 6: e595–600. 38987117 10.1016/S2589-7500(24)00114-6 PMC11299190

[30] Haghighi T, Gholami S, Sokol JT, EYE-Llama, an in-domain large language model for ophthalmology. bioRxiv 2024. DOI:10.1101/2024.04.26.591355. PMC12281063 40697409

[31] Singer MB, Fu JJ, Chow J, Teng CC. Development and Evaluation of Aeyeconsult: A Novel Ophthalmology Chatbot Leveraging Verified Textbook Knowledge and GPT-4. Journal of Surgical Education 2024; 81: 438–43. 38135548 10.1016/j.jsurg.2023.11.019

[32] Chen X, Zhao Z, Zhang W, EyeGPT Ophthalmic Assistant with Large Language Models. 2024. httpsarxiv.orgabs2403.00840.

[33] de Hond A, Leeuwenberg T, Bartels R, From text to treatment: the crucial role of validation for generative large language models in health care. The Lancet Digital Health 2024; 6: e441–3. 38906607 10.1016/S2589-7500(24)00111-0

[34] Zhao Z, Jin Q, Chen F, Peng T, Yu S. Pmc-patients: A large-scale dataset of patient summaries and relations for benchmarking retrieval-based clinical decision support systems. arXiv preprint arXiv:220213876 2022. 10.1038/s41597-023-02814-8 PMC10728216 38110415

[35] Keloth VK, Hu Y, Xie Q, Advancing entity recognition in biomedicine via instruction tuning of large language models. Bioinformatics 2024; 40: btae163. 38514400 10.1093/bioinformatics/btae163 PMC11001490

[36] Agrawal M, Hegselmann S, Lang H, Kim Y, Sontag D. Large Language Models are Few-Shot Clinical Information Extractors. 2022. https://arxiv.org/abs/2205.12689.

[37] Chen Q, Du J, Hu Y, Large language models in biomedical natural language processing: benchmarks, baselines, and recommendations. 2024. https://arxiv.org/abs/2305.16326.

[38] Touvron H, Martin L, Stone K, Llama 2: Open Foundation and Fine-Tuned Chat Models. 2023. https://arxiv.org/abs/2307.09288.

[39] Raiaan MAK, Mukta MdSH, Fatema K, A Review on Large Language Models: Architectures, Applications, Taxonomies, Open Issues and Challenges. IEEE Access 2024; 12: 26839–74.

[40] Minaee S, Mikolov T, Nikzad N, Large Language Models: A Survey. 2024. https://arxiv.org/abs/2402.06196.

[41] Hu JE, Shen Y, Wallis P, LoRA: Low-Rank Adaptation of Large Language Models. ArXiv 2021; abs/2106.09685.

[42] American Academy of Ophthalmology. Ask an Ophthalmologist. 2024. https://www.aao.org/eye-health/ask-ophthalmologist.

[43] Cohan A, Dernoncourt F, Kim DS, A Discourse-Aware Attention Model for Abstractive Summarization of Long Documents. 2018. https://arxiv.org/abs/1804.05685.

[44] Koh HY, Ju J, Liu M, Pan S. An Empirical Survey on Long Document Summarization: Datasets, Models, and Metrics. ACM Comput Surv 2022; 55. DOI:10.1145/3545176.

[45] Tran H, Yang Z, Yao Z, Yu H. BioInstruct: instruction tuning of large language models for biomedical natural language processing. Journal of the American Medical Informatics Association 2024; : ocae122. 10.1093/jamia/ocae122 PMC11339494 38833265

[46] Lim ZW, Pushpanathan K, Yew SME, Benchmarking large language models’ performances for myopia care: a comparative analysis of ChatGPT-3.5, ChatGPT-4.0, and Google Bard. eBioMedicine 2023; 95. DOI:10.1016/j.ebiom.2023.104770. PMC10470220 37625267

[47] He Z, Wang Y, Yan A, MedEval: A Multi-Level, Multi-Task, and Multi-Domain Medical Benchmark for Language Model Evaluation. 2023. https://arxiv.org/abs/2310.14088.

[48] Liu Z, Li Y, Shu P, Radiology-Llama2: Best-in-Class Large Language Model for Radiology. 2023. https://arxiv.org/abs/2309.06419.

[49] Antaki F, Touma S, Milad D, El-Khoury J, Duval R. Evaluating the Performance of ChatGPT in Ophthalmology: An Analysis of Its Successes and Shortcomings. Ophthalmology Science 2023; 3. DOI:10.1016/j.xops.2023.100324. PMC10272508 37334036

[50] Antaki F, Milad D, Chia MA, Capabilities of GPT-4 in ophthalmology: an analysis of model entropy and progress towards human-level medical question answering. British Journal of Ophthalmology 2023. DOI:10.1136/bjo-2023-324438. 37923374

[51] Lee P, Goldberg C, Kohane I. The AI revolution in medicine: GPT-4 and beyond. Pearson, 2023.

[52] Nori H, Lee YT, Zhang S, Can generalist foundation models outcompete special-purpose tuning? case study in medicine. arXiv preprint arXiv:231116452 2023.

[53] Hadi MU, Qureshi R, Shah A, Large language models: a comprehensive survey of its applications, challenges, limitations, and future prospects. Authorea Preprints 2023.

[54] Singhal K, Azizi S, Tu T, Large language models encode clinical knowledge. Nature 2023; 620: 172–80. 37438534 10.1038/s41586-023-06291-2 PMC10396962

