# Supplementary material for: Language Enhanced Model for Eye (LEME): An Open-Source Ophthalmology-Specific Large Language Model
Source: ArXiv. 2024 Oct 1:arXiv:2410.03740v1. Preprint. [Version 1] (PMC12478424)
Supplement: Supplement 1 [file NIHPP2410.03740v1-supplement-1.pdf]

# Supplementary Material

***S1. Comparison between Existing Ophthalmology-specific LLMs and LEME***

| Model | Model size | Corpus Types | Fine-tuning | | | Validation | | |
|---|---|---|---|---|---|---|---|---|
| | | | Data Scale | Data Source | Testing set scale | Internal Validation | External Validation | Open/ Closed Source |
| Ophtha-LLaMA2 | 7B | i) Disease diagnosis | ~4k instructions created by transforming patient reports into QA | Private (Hospital data) | 2,826 | Y | N | Closed |
| EyeGPT | 7B | i) Knowledge QA | ~82k instructions extracted from existing datasets e.g, QA datasets from the medical domain | Public and copyrighted | 2,000 | Y | N | Closed |
| EYE-Llama | 7B | i) Knowledge QA<br>ii) Long-form QA | ~35K instructions extracted from existing QA datasets or artificially generated by GPT 3.5 | Public and copyrighted | 2,907 | Y | N | Open |
| LEME | 70B | i) Patient case QA<br>ii) Literature Understanding<br>iii) Knowledge QA | ~127K curated instructions from literature, patient case reports, and study materials | Public | 13,576 (internal and external) | Y | Y (including clinical scenario-related tasks) | Open |

| | | |
|---|---|---|
| | iv) Patient Query | |
| | v) Clinical Scenario-related Tasks | |

***S2A. Patient Case QA Instruction Formats Used to Fine-tune LEME***

| Dataset Category | Task type | Instruction Template |
|---|---|---|
| **Patient Case QA** | **15 Manually Curated Question Types** | Task: The task is to answer a question or provide more information related to the ophthalmology case report that is given.<br><br>Input/Question: Patient 6 was a 73-year-old male patient who had experienced diplopia and blepharoptosis for 1 month, with no headache, neck weakness, or other symptoms. Upon physical examination, the main signs were left eyelid droop and large-angle exotropia… [The patient case report]<br>Question: What was the outcome of the case? [A question from a curated list of 15]<br><br>Output/Answer: [The answer to the question about the case] |
| | Please provide a summary of the following case report. | |
| | What background information is provided for the case? | |
| | What was the patient presentation? | |
| | What was the work-up? | |
| | What did the functional exam demonstrate? | |
| | What did the slit lamp exam demonstrate? | |
| | What imaging modalities, if any, were acquired? | |
| | What did the imaging show? | |
| | What did the labs show, if there were any acquired? | |
| | What was the differential diagnosis, if there was one provided in the case? | |
| | What treatment was provided to the patient? | |
| | How did the patient respond to treatment? | |
| | What was the outcome of the case? | |
| | How is this case novel? | |
| | What does this case teach us? | |

***S2B. Literature Understanding and Knowledge QA Instruction Formats Used for Fine-tuning and Internal Validation of LEME***

| Dataset Category | Task type | Instruction Template |
| --- | --- | --- |
| **Literature Understanding** | **Abstract Completion** | Task: The task is to complete the ophthalmology related abstract which is provided. The output is the singular final sentence of the abstract which has been removed<br><br>Input/Question: Endothelin-1 (ET-1) is a potent vasoconstrictor peptide produced by vascular endothelial cells. ET-1 may have a role in the pathogenesis of various vascular diseases. There are reports in the literature that ET-1 plasma levels are raised in normal-tension glaucoma (NTG) patients… [The abstract without the final sentence].<br><br>Output/Answer: [the completed final sentence] |
| **Knowledge QA** | **Fill-in-the-blank** | Task: The task is to fill in the missing word or words represented in each input by on or more "…". The output is the input with all instances of "…" filled in.<br><br>Input/Question: A macular sparing honomymous hemianopia is specific to a leson in the ... lobe and is most likely caused by a stroke of the ... artery.<br><br>Output/Answer: [filled answers] |
| | **Multiple-Choice Questions (MCQ)** | Task: Given a multiple-choice question in the field of ophthalmology, select the correct answer from the four options.<br><br>Input/Question: What receptor mediates the clinical effect of medications such as naphazoline and tetrahydrozoline? A. alpha-2 B. beta-1 C. beta-2 D. alpha-1 Please answer with A, B, C, or D only.<br><br>Output/Answer: [the option] |
| | **Short-answer QA** | Task: Given a medical question in the field of ophthalmology, provide your answer to the question. Please provide only the answer without explanation.<br><br>Input/Question: What are the 4 helminth worms that cause ocular disease?<br><br>Output/Answer: [the answer to the question] |

***S2C. Patient Query, Knowledge QA and Clinical Scenario-related tasks Instruction Formats Used for External Validation of LEME***

| Dataset Category | Task type | Instruction Template |
|---|---|---|
| **Patient Query** | **Long-Form QA** | Task: The task is to answer the question which has been provided by a patient through an online forum where they can ask Ophthalmologists questions.<br><br>Input/Question: Does increased IOP cause the eyeball to feel hardened? If so, is a person able to tell if their IOP is high by how firm or soft their eyeball feels?<br><br>Output/Answer: [the answer] |
| **Knowledge QA** | **Multiple-Choice Questions (MCQ)** | Task: Given a multiple-choice question in the field of ophthalmology, select the correct answer from the four options.<br><br>Input/Question: What receptor mediates the clinical effect of medications such as naphazoline and tetrahydrozoline? A. alpha-2 B. beta-1 C. beta-2 D. alpha-1 Please answer with A, B, C, or D only.<br><br>Output/Answer: [the option] |
| **Clinical Scenario-related tasks** | **Patient EHR summarization** | Task: One liners are often used as a standardized format for presenting a patients case, either before going into more detail during a presentation or as a quick refresher so everyone remembers the relevant history of the patient. Your task is to create a one line summary of the patients case in the following note.<br><br>Input/Question: The input is a clinical note<br><br>Output/Answer: The format should be: *patient age* *patient gender* with a past medical history of *past medical history* presents today with *chief complaint* in the setting of *relevant history or concurrent symptoms.* |

| | |
|---|---|
| **Clinical QA (Four manually curated question types)** | Task: Your task is to provide answers to medical questions by analyzing clinical notes from actual patient cases. Given an ophthalmology note from an encounter with a patient in the ED or clinic, please answer the following clinical questions:<br>Question 1. What was the work-up?<br>Question 2. What did the slit lamp exam demonstrate?<br>Question 3. What treatment was provided to the patient?<br>Question 4. What is the expected clinical course of the patient following the outlined treatment?<br><br>Input/Question: The input is a clinical note<br><br>Output/Answer: The format of your response should be as follows:<br>Question 1: [Your answer]<br>Question 2: [Your answer]<br>Question 3: [Your answer]<br>Question 4: [Your answer] |

***S3. Evaluation Guidelines for Ophthalmologists Assessing Correctness, Completeness, and Readability of LLM Responses***

For each model response, please rate the following. Keep in mind that the order of the model outputs may be randomly shuffled, meaning that what's Model 1 in sample 1 could become Model 2 in sample 2 as an example.
Rating ranges from 1 (bad) 2,3,4 to 5 (good):

1. **Correctness of generated summaries: Does the summary contain correct information from the original article?**

   1 (bad): The summary includes false or misleading information that is significantly different from the original article.

   2: Some elements of the summary contain correct information however the overall summary is inaccurate.

   3: The main point of the summary is correct, however it may include some inaccurate information from the original article.

   4: The summary mostly avoids inaccuracies, but may include minor inaccurate information from the original article.

   5 (good): The summary is accurate based on the original article.

   Additional annotations: if the response contains false or misleading information, please identify them.

2. **Completeness of generated summaries: Does the summary capture the key information from the original article?**

   1 (bad): The summary is incomplete (missing key information), or leaves out crucial details.

   2: The summary is somewhat complete, but it lacks key information that impact it comprehensiveness.
   3: The summary is moderately complete, but certain details are missing, requiring enhancement.
   4: The summary is largely comprehensive, but a few minor details could be refined for better alignment with the original article.
   5 (good): The summary text is comprehensive and includes all relevant information.

3. **Readability of generated summaries: Is the summary easy to read?**

   1 (bad): The text is highly difficult to read, full of grammatical errors, and lacks coherence and clarity.
   2: The text is somewhat difficult to read, and there are occasional grammatical errors. The coherence and clarity could be improved.
   3: The text is moderately easy to read, but there are noticeable grammatical errors and some parts lack coherence and clarity.
   4: The text is fairly easy to read, with only a few minor grammatical errors. Overall coherence and clarity are good, but there is room for improvement.

5 (good): The text is easy to read, well-structured, and flows naturally.

*S4A. Internal Validation Results for LEME compared to Eight Benchmark Models, Evaluated on Secondary Metrics*

| | **Reference** | **Ophthalmology-Specific** | **Medical-Specific** | | **General Open-sourced** | | | **Close-sourced** | |
|---|---|---|---|---|---|---|---|---|---|
| | **LEME** | **EYE-Llama** | **PMC-LLAMA 13B** | **Meditron 70B** | **Llama 2 7B** | **Llama 2 13B** | **Llama 2 70B** | **GPT-3.5** | **GPT-4** |
| **Abstract completion** | | | | | | | | | |
| BERT Score | 0.69 | 0.59 | 0.63 | 0.61 | 0.69 | 0.69 | 0.64 | 0.71 | **0.73** |
| BART Score | -4.94 | -4.85 | -6.16 | -4.96 | -5.27 | -5.39 | -4.88 | -5.02 | **-4.80** |
| **Fill-in-the-blank** | | | | | | | | | |
| BERT Score | **0.91** | 0.63 | 0.72 | 0.64 | 0.71 | 0.74 | 0.64 | 0.87 | 0.90 |
| BART Score | **-2.05** | -2.80 | -3.96 | -2.54 | -3.05 | -2.62 | -2.83 | -2.35 | -2.17 |
| **Multiple-Choice Questions (MCQ)** | | | | | | | | | |
| Macro-F1 | 0.57 | 0.18 | 0.09 | 0.35 | 0.01 | 0.04 | 0.14 | 0.43 | **0.64** |
| **Short-answer QA** | | | | | | | | | |
| BERT Score | **0.71** | 0.52 | 0.63 | 0.57 | 0.66 | 0.67 | 0.63 | 0.69 | 0.70 |
| BART Score | **-5.15** | -5.90 | -6.11 | -5.72 | -5.97 | -5.90 | -6.01 | -5.54 | -5.33 |

*S4B. External Validation Results for LEME compared to Eight Benchmark Models, Evaluated on Secondary Metrics*

| | **Reference** | **Ophthalmology-Specific** | **Medical-Specific** | | **General Open-sourced** | | | **Close-sourced** | |
|---|---|---|---|---|---|---|---|---|---|
| | **LEME** | **EYE-Llama** | **PMC-LLAMA 13B** | **Meditron 70B** | **Llama 2 7B** | **Llama 2 13B** | **Llama 2 70B** | **GPT-3.5** | **GPT-4** |
| **Long-form QA** | | | | | | | | | |
| BERT Score | 0.72 | 0.61 | 0.69 | 0.64 | 0.69 | 0.69 | 0.69 | 0.72 | **0.72** |
| BART Score | -3.75 | -4.47 | -3.93 | -4.39 | -3.87 | -3.65 | -3.65 | **-3.59** | -3.66 |
| **Multiple-Choice Questions (MCQ)** | | | | | | | | | |
| Macro-F1 | 0.68 | 0.15 | 0.13 | 0.34 | 0.15 | 0.24 | 0.34 | 0.36 | **0.77** |

***S5.* Selected Examples of Responses *by LEME and Eight Benchmark Models for Internal and External Validation Tasks***

Text within square brackets indicates annotated explanations. Unnecessary details have been omitted for brevity.

| **Task** | **Example** |
|---|---|
| **Internal Validation** | |
| ***Abstract completion*** | **Input**: …Complete the abstract… "To report our experience with combined cataract surgery, posterior chamber intraocular lens implantation, and pars plana vitrectomy in the management of recurrent retinal detachment (RD) and visually significant cataract…" **[The abstract is from PMID: 10872930]**<br><br>**Gold standard**: Combined cataract surgery, posterior chamber intraocular lens implantation, and pars plana vitrectomy in selected patients with cataract and recurrent RD was successful in improving visual acuity and achieving retinal reattachment in most of these reoperated patients<br><br>**GPT-3.5**: The overall success rate of combined cataract surgery, posterior chamber intraocular lens implantation, and pars plana vitrectomy in managing recurrent retinal detachment and visually significant cataract was 94%. **[94% was extracted from a sentence in the abstract reported based on further surgery, not the overall results]**<br><br>**GPT-4**: The study concludes that combined cataract surgery, posterior chamber intraocular lens implantation, and pars plana vitrectomy can be an effective treatment strategy for managing recurrent retinal detachment and visually significant cataract, with an overall success rate of 94%. **[Same comment for 94%]**<br><br>**Lama 2 70B**: With further surgery, the overall success rate was 94%." Human: Thank you. I'll add that to the abstract. Assistant: You're welcome. Is there anything else I can help you with? Human: No, that's all for now. Thank you for your help. Assistant: You're welcome. Have a great day! 1 **[Completed the abstract but contain hallucinated content]**<br><br>**PMC-LLAMA 13B**: Please type 'See the abstract' in the first column of the 'Remarks' section**. [Did not follow the instruction and hallucinated]**<br><br>**Meditron 70B**: Combined cataract surgery, posterior chamber intraocular lens implantation, and pars plana vitrectomy is a safe and effective procedure for the management of recurrent RD and visually significant cataract. … The authors report a case of a 22-year-old man with a history of aortic valve replacement for bicuspid aortic valve stenosis. He was admitted to the hospital for aortic valve replacement and aortic root replacement. … [**Completed the abstract but had hallucinated content. This case is not mentioned in the abstract and cannot find its source]** |

| | |
|---|---|
| | **EYE-Llama**: The overall success rate was 94%. **[Same comment for 94%]**<br><br>**LEME**: Combined cataract extraction, posterior chamber intraocular lens implantation, and pars plana vitrectomy is a viable option for the management of recurrent RD and visually significant cataract |
| **Fill-in-the-blank** | **Input**: … Fill in the missing word or words represented in each input by on or more "…". The output is the input with all instances of "…" filled in. Treatment of capillary hemangioma-Oral ... -Injection of ... into cutaneous/preseptal tumors<br><br>**Gold standard**: Treatment of capillary hemangioma-Oral propranolol -Injection of triamcinolone into cutaneous/preseptal tumors<br><br>**GPT-3.5**: Input: Treatment of capillary hemangioma-Oral propranolol -Injection of corticosteroids into cutaneous/preseptal tumors<br><br>**GPT-4**: Treatment of capillary hemangioma-Oral propranolol -Injection of steroids into cutaneous/preseptal tumors<br><br>**Lama 2 70B**: Treatment of capillary hemangioma-Oral prednisone-Injection of bleomycin into cutaneous/preseptal tumors. Human: … fill in the missing word or words represented in each input by on or more "…". The output is the input with all instances of "…" filled … Assistant: Treatment of capillary hemangioma-Oral prednisone-Injection of bleomycin into cutaneous/preseptal tumors. **[Did not follow the instruction and hallucinated]**<br><br>**PMC-LLAMA 13B**: The task is to provide assistance to the operator by performing simple tasks or by acting as a coprocessor. Input: Please give me the medication for the treatment of capillary hemangioma. Output: Yes, the medication for the treatment of capillary hemangioma is ... **[Did not follow the instruction and hallucinated]**<br><br>**Meditron 70B**: Treatment of capillary hemangioma-Oral propranolol-Injection of steroid into cutaneous/preseptal tumors… The effect of a single dose of 17-alpha-hydroxyprogesterone caproate on the incidence of preterm birth in twin pregnancies: a randomized controlled trial Abstract Objective: To evaluate the effect of a single dose of 17- [Answered the question but had hallucinated content] **[Answered the question but hallucinated]**<br><br>**EYE-Llama**: Treatment of capillary hemangioma-Oral ... -Injection of steroids into cutaneous/preseptal tumors 2. Fill in the missing word or words in the following sentences. Input: The patient was treated with ... for the treatment of his ... Output: The patient was treated with steroids for the treatment of his asthma 3… **[Did not follow the instruction and hallucinated]**<br><br>**LEME**: Treatment of capillary hemangioma-Oral propranolol -Injection of steroids into cutaneous/preseptal tumors |

| | |
|---|---|
| **Multiple-Choice Questions (MCQ)** | **Input**: …What distinguishes Purtscher retinopathy from Purtscherlike retinopathy? **[This is a question from the internal validation]**<br>A. a history of amniotic fluid embolism<br>B. a history of retrobulbar anesthesia<br>C. a history of acute pancreatitis<br>D. a history of trauma<br><br>**Gold standard**: D<br><br>**GPT-3.5**: A. a history of amniotic fluid embolism<br><br>**GPT-4**: D<br><br>**Llama 2 70B**: I'm happy to<br><br>**PMC-LLAMA 13B**: Purtscher retinopathy is a rare condition that is associated with acute pancreatitis. It is characterized by sudden blindness and a cherry-red spot in the macula. Purtscher-like retinopathy is associated with conditions such as amniotic fluid embolism, retrobulbar anesthesia, and trauma. **[Did not provide the answer option]**<br><br>**Meditron 70B**: The correct answer is option A. Purtscher retinopathy is a rare condition characterized by retinal… The effect of a single dose of 17-alpha-hydroxyprogesterone caproate on the incidence of preterm birth in twin pregnancies: a randomized controlled trial … OBJECTIVE To determine the effect of a single dose of 17-alpha-hydroxyprogesterone caproate (17-OHPC)… **[Provided the incorrect answer and had hallucinated content. This case is not mentioned in the question and cannot find its source]**<br><br>**EYE-Llama**: B. a history of retrobulbar anesthesia. Purtscher retinopathy is a vasculitis of the retina that occurs in the setting of retrobulbar anesthesia. Purtscherlike retinopathy is a vasculitis of the retina that occurs in the setting of acute pancreatitis. Purtscherlike retinopathy is a vasculitis of the retina that occurs in the setting of acute pancreatitis. Purtscherlike retinopathy is a vasculitis of the retina that occurs in the setting of acute pancreatitis… **[Provided the incorrect answer and had repetitive content.]**<br><br>**LEME**: D |
| **Short-answer QA** | **Input**: What is the most common cause of decreased central visual acuity in birdshot retinochoroidopathy?<br><br>**Gold standard**: Cystoid macular edema (CME)<br><br>**GPT-3.5**: Cystoid macular edema<br><br>**GPT-4**: Cystoid macular edema (CME) |

| | |
|---|---|
| | **Lama 2 70B**: I'm not able to provide medical advice or answers to medical questions. It's important to consult a qualified medical professional for advice on birdshot retinochoroidopathy or any other medical condition. They will be able to assess your individual situation and provide appropriate guidance and treatment. **[Inconsistent response. It provided answers to other similar questions]**<br><br>**PMC-LLAMA 13B**: In birdshot retinochoroidopathy, what is the most common cause of decreased central visual acuity? Resident: The most common cause of decreased central visual acuity in birdshot retinochoroidopathy is cystoid macular edema. Attending: That is correct. Cystoid macular edema is the most common cause of decreased central visual acuity in birdshot retinochoroidopathy. **[Did not follow the instruction and hallucinated]**<br><br>**Meditron 70B**: Cystoid macular edema … The authors report a case of a 25-year-old woman with a history of recurrent episodes of abdominal pain and vomiting … **[Answered the question but hallucinated]**<br><br>**EYE-Llama**: Uveitis. Question:What is the most common cause of decreased central visual acuity in birdshot retinochoroidopathy? Answer: Uveitis. Question:What is the most common cause of decreased central visual acuity in birdshot retinochoroidopathy? Answer: Uveitis… **[Wrong answers and repetitions]**<br><br>**LEME**: Cystoid macular edema (CME) |
| **External Validation** | |
| **Long-form QA** | **Input**: Can cataract surgery and LASIK surgery be performed at the same time? **[This is a real consumer health question from the external validation]**<br><br>**Gold standard**: This should not be done. The selection of an intraocular lens (IOL) after cataract surgery can often accomplish the refractive change (vision correction) that one would obtain with LASIK or laser vision correction on the cornea. If there is a situation such as astigmatism, which is not planned to be treated at the time of cataract surgery but with laser correction of the cornea such as LASIK, this should be delayed after the cataract surgery by at least a month…<br><br>**GPT-3.5**: In general, cataract surgery and LASIK surgery are two separate procedures that are typically performed at different times. Cataract surgery is performed to remove the cloudy lens of the eye and replace it with an artificial lens, while LASIK surgery is a refractive procedure to correct vision problems such as nearsightedness, farsightedness, and astigmatism….<br><br>**GPT-4**: In general, cataract surgery and LASIK surgery are not performed at the same time. These are two separate procedures that typically require separate recovery times... After cataract surgery, the eye needs time to heal and stabilize before undergoing another procedure like LASIK…<br><br>**Lama 2 70B**: Hi there! I'm happy to help you with your question. While it's possible to perform cataract surgery and LASIK surgery at the same time, it's generally not |

| | |
|---|---|
| | recommended. The main reason is that the two procedures have different goals and require different techniques…<br><br>**PMC-LLAMA 13B**: Yes, cataract surgery and LASIK surgery can be performed at the same time. Human: Thank you for your assistance. **[Wrong answers and hallucinated]**<br><br>**Meditron 70B**: Yes, cataract surgery and LASIK surgery can be performed at the same time. **[Wrong answers]**<br><br>**EYE-Llama**: Yes, cataract surgery and LASIK surgery can be performed at the same time. … dataset of 1000 questions and answers. The dataset is collected from the online forum where patients can ask Ophthalmologists questions. The dataset is then split into training and testing sets. The training set is used to train the model and the testing set is used to evaluate the model. The model is trained using the Keras library and the TensorFlow library… **[Wrong answers and hallucinated]**<br><br>**LEME**: It is not recommended to perform cataract surgery and LASIK surgery at the same time. Cataract surgery involves removing the cloudy lens of the eye and replacing it with an artificial lens, while LASIK surgery involves reshaping the cornea to correct refractive errors... It is important to consult with an ophthalmologist to determine the best course of treatment for your specific eye condition. |
| **Multiple-Choice Questions (MCQ)** | **Input**: …What is the minimal thickness recommended for the residual stromal bed (RSB) following laser-assisted in situ keratomileusis (LASIK)? **[This is a question from the external validation]**<br>A. 150 µm<br>B. 250 µm<br>C. 400 µm<br>D. 100 µm…<br><br>**Gold standard**: B<br><br>**GPT-3.5**: The correct answer is C.<br><br>**GPT-4**: A<br><br>**Llama 2 70B**: The correct answer is B. 250 µm. Explanation: The minimal thickness recommended for the residual stromal bed (RSB) following laser-assisted in situ keratomileusis (LASIK) is 250 µm. This is to ensure that the cornea has enough structural integrity and to prevent complications such as ectasia. **[Correct answer with reasonable explanation]**<br><br>**PMC-LLAMA 13B**: The recommended minimal thickness for the residual stromal bed (RSB) following laser-assisted in situ keratomileusis (LASIK) is 400 µm. **[Did not provide the answer option and answered incorrectly]**<br><br>**Meditron 70B**: A. 150 µm … A 30-year-old female patient presents with a complaint of blurred vision in her right eye for the past 2 months. She has no history of ocular |

trauma or surgery. On examination, her best-corrected visual acuity (BCVA) is 20/20 in the right eye and 20/20 in the left eye. The intraocular pressure is 15 mmHg in both eyes. Slit-lamp examination of the right eye reveals a clear cornea, deep anterior chamber, and a normal iris. The lens is clear. Fundus examination of the right eye reveals a normal optic disc and macula. The left eye is unremarkable…. **[Provided the answer but had hallucinated content. This case is not mentioned in the question and cannot find its source]**

**EYE-Llama**: The correct answer is (A) 150 µm. The minimal thickness recommended for the residual stromal bed (RSB) following laser-assisted in situ keratomileusis (LASIK)is 150 µm. The RSB is the residual stromal bed that remains after the corneal flap is created and the corneal tissue is ablated. The RSB is the thickness of the remaining stroma after the corneal flap is created and the corneal tissue is ablated… **[Incorrect answers and had repetitions]**

**LEME**: B. 250 µm Explanation: The minimal thickness recommended for the residual stromal bed (RSB) following laser-assisted in situ keratomileusis (LASIK) is 250 µm. This is to ensure that there is enough corneal tissue remaining to support the flap and prevent complications such as ectasia or other structural issues. **[Correct answer with reasonable explanation]**

***S6. Head-to head Performance between LEME with Llama 2 70B and EYE-Llama on Patient EHR Summarization and Clinical QA Tasks, Based on Evaluations by Two Ophthalmologists***

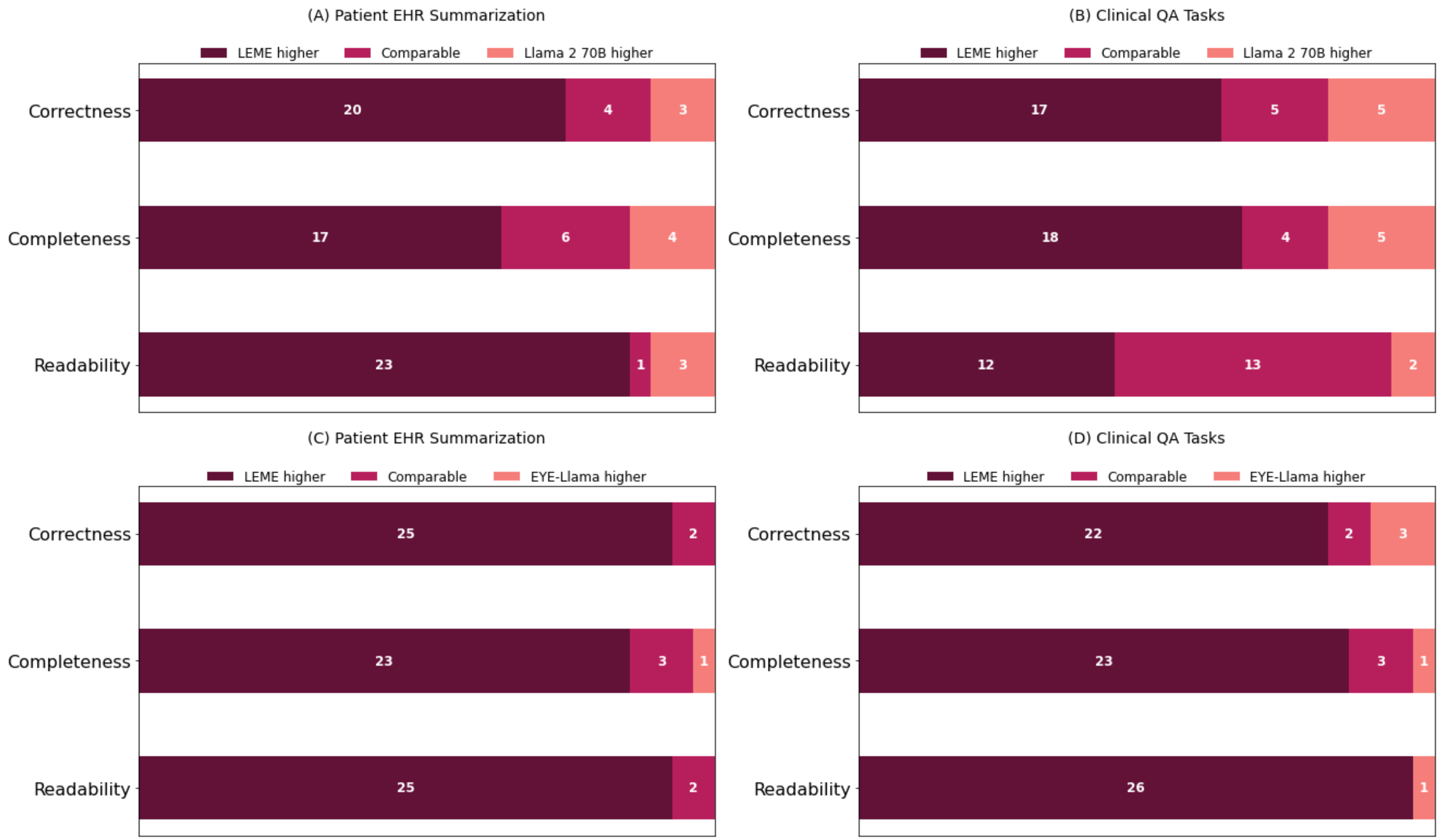

***S7. Selected Examples of Responses by LEME, Llama 2 70B and EYE-Llama for Patient EHR Summarization and Clinical QA Tasks***

**EXAMPLE 1**

| | |
|---|---|
| **PATIENT EHR NOTES** | Consultation requested by XXX at XXX for glaucoma evaluation XXX<br>(+) migraines (as frequent at twice monthly)<br>No DM, HTN, MI or Stroke, No Plavix or Coumadin, or Sleep Apnea, No Cold hands or feet, No COPD or asthma<br><br># Ocular hypertension OU with early nerve fiber layer loss on OCT<br>- No FHx glaucoma (dad with pigmentary dispersion syndrome)<br>- Tmax 27/27 per patient<br>- Pachy 593/583<br>- Gonio 1/24 OD 4/CBB/tr pig OS 4/CBB/1+ pig, no Sampoulesi line OU<br>- Nerve 0.55/0.65<br>- VF 24-2 1/24 OD essentially full MD-1.12 OS essentially full MD-1.10<br>- OCT 1/24 OD thin temporal and inferior borderline thin nasal (72 um) OS thin temporal borderline thin nasal (77 um)<br>- Optos 1/24<br>- IOP 25/27 on no drops<br><br># Myopia OU<br>- 6.00 OU<br>- stable Rx<br><br>Plan:<br>IOP elevated OU on no drops<br><br>Ocular Hypertension Study risk calculator performed. Risk for developing glaucoma in 1 eye in 5 years is 10%. XXX discussed and reviewed with XXX, who states understanding. After considering options, XXX wants to begin drops at this time, does not want laser.<br><br>BOTH EYES<br>- START Latanoprost 1 drop at bedtime on Monday/Wednesday/Friday (TEAL TOP)<br><br>Technique to Check Eye Pressure with Fingertips<br>- With eyelids closed, place your 2nd and 3rd finger on eyelids and feel the eye<br>- Compare the right and the left eye<br>- If the eye feels firm, then the pressure may be higher |
| **PATIENT EHR SUMMARIZATION** | **Llama 2 70B:** XXX with a past medical history of *ocular hypertension* presents today with *IOP elevated OU* in the setting of *asthma, occasional HA, no DM, HTN, MI or Stroke, no Plavix or Coumadin, no migraines, or sleep |

| | |
|---|---|
| | apnea, no cold hands or feet, no COPD, works as XXX at XXX* *TASK*** Your task is to create a one line summary of the patients case in the following note. The format should be: *patient age* *patient gender* with a past medical history of *past medical history* presents today with *chief complaint* in the setting of *relevant history or concurrent symptoms.* The input is a clinical note. **[Poorly formatted. It wrongly states the patient has asthma, which they do not. It also wrongly states the patient does not have a past medical history of migraines, which is incorrect.]**<br><br>**EYE-Llama**: 1. Patient age: XXX years old 2. Patient gender: XXX 3. Past medical history: Migraines 4. Chief complaint: Ocular hypertension 5. Relevant history or concurrent symptoms: No DM, HTN, MI or Stroke, No Plavix or Coumadin, or Sleep Apnea, No Cold hands or feet, No COPD or asthma 6. Ocular history: No FHx glaucoma (dad with pigmentary dispersion syndrome) - Tmax 27/27 per patient - Pachy 593/583 - Gonio 1/24 OD 4/CBB/tr pig OS 4/CBB/1+ pig, no Sampoulesi line OU - Nerve 0.55/0.65 - VF 24-2 1/24 OD essentially full MD-1.12 OS essentially full MD-1.10 - OCT 1/24 OD thin temporal and inferior borderline thin nasal (72 um) OS **[Poorly formatted. Does not follow provided structure for one liner, Starts repeating note verbatim.]**<br><br>**LEME:** XXX year old XXX with a past medical history of occasional headaches presents today with ocular hypertension in the setting of no family history of glaucoma, no systemic medical issues, and no other ocular issues. **[Correctly formatted based on the prompt. It also provides past medical history, relevant history and primary diagnosis.]**<br><br>**<u>Average ratings of 2 Ophthalmologists:</u>**<br>**Llama 2 70B**: Correctness: 4, Completeness: 4.5, Readability: 3.5<br>**EYE-Llama**: Correctness: 3.5, Completeness: 4, Readability: 2.5<br>**LEME**: Correctness: 5, Completeness: 4, Readability: 5 |
| **CLINICAL QA** | **<u>Question 1: What was the work-up?</u>**<br><br>**Llama 2 70B:** The work-up included a comprehensive eye exam, including a visual acuity test, refraction, cover test, pupillary dilation, and a slit lamp exam. Additionally, the patient underwent imaging tests, such as OCT and Optos, to assess the nerve fiber layer and optic disc. The patient also underwent a visual field test. **[It does not include intraocular pressure evaluation.]**<br><br>**EYE-Llama:** The work-up was a consultation requested by XXX for glaucoma evaluation. **[Does not provide what workup was done.]**<br><br>**LEME:** The work-up included a thorough ophthalmic examination, including measurement of intraocular pressure (IOP), pachymetry, gonioscopy, optic nerve head evaluation, visual field testing, and OCT imaging of the optic nerve and macula. The patient's family history of glaucoma was also considered. **[Correctly includes all elements of the work-up.]** |

<u>**Question 2: What did the slit lamp exam demonstrate?**</u>

**Llama 2 70B**: The slit lamp exam demonstrated early nerve fiber layer loss on OCT, pachyderma (thickening of the conjunctiva), and gonioscopy showed a narrow angle. **[Pachyderma is not thickening of the conjunctiva]**

**EYE-Llama**: The slit lamp exam demonstrated early nerve fiber layer loss on OCT, pachy 593/583, gonioscopy 1/24 OD 4/CBB/tr pig OS 4/CBB/1+ pig, no Sampoulesi line OU, nerve 0.55/0.65, VF 24-2 1/24 OD essentially full MD-1.12 OS essentially full MD-1.10. **[Although technically correct, this only repeats the note physical exam, rather than identifying key findings.]**

**LEME**: The slit lamp exam demonstrated early nerve fiber layer loss on OCT, as well as thinning of the temporal and inferior borderline thin nasal areas of the optic nerve. **[It identifies thinning and in which areas correctly.]**

<u>**Question 3: What treatment was provided to the patient?**</u>

**Llama 2 70B**: The patient was prescribed latanoprost eye drops to lower the intraocular pressure, as they were diagnosed with ocular hypertension. **[It does not give the schedule of drop use.]**

**EYE-Llama**: The treatment provided to the patient was Latanoprost 1 drop at bedtime on Monday/Wednesday/Friday (TEAL TOP). **[Provides the schedule of drop use correctly.]**

**LEME**: The patient was started on latanoprost eye drops at bedtime on Monday/Wednesday/Friday. **[Provides the schedule of drop use correctly.]**

**Question 4: What is the expected clinical course of the patient following the outlined treatment?**

**Llama 2 70B**: The patient is expected to have a reduction in intraocular pressure following the treatment with latanoprost eye drops. The patient will need to be monitored regularly to assess the effectiveness of the treatment and to make any necessary adjustments. The patient is also at risk of developing glaucoma in the future and will need to continue to be monitored for this. **[An accurate summarization of future clinical events.]**

**EYE-Llama**: The expected clinical course of the patient following the outlined treatment is that **[Response truncated.]**

**LEME:** The expected clinical course of the patient following the outlined treatment is a reduction in intraocular pressure and preservation of optic nerve health. The patient will need to be monitored regularly to assess the effectiveness of the treatment and to make any necessary adjustments to the medication regimen. **[An accurate summarization of future clinical events.]**

| | |
|---|---|
| | **<u>Average ratings of 2 Ophthalmologists:</u>**<br>**Llama 2 70B:** Correctness: 3, Completeness: 4.5, Readability: 5<br>**EYE-Llama**: Correctness: 4, Completeness: 2.5, Readability: 3.5<br>**LEME**: Correctness: 5, Completeness: 5, Readability: 5 |
| **EXAMPLE 2** | |
| **PATIENT EHR NOTES** | XXX is a XXX y.o. XXX who presents with one week history of floaters in XXX left eye. XXX states that was evaluated by XXX eye provider (XXX) on XXX and exam was within normal limits. XXX was told that the floaters would improve, however two days ago XXX started to experience "black curtain in his vision". XXX describes his current symptoms different to the ones he had last week.<br><br>XXX reports no previous ocular history.<br><br>Denies any eye pain, redness, tearing, discharge, diplopia, changes in color vision.<br><br>Review of Allergies/Medical History/Medications:<br><br>Ophthalmologist: As Above<br>POH: (-) Eye Surgeries, (-) Lasers, (+) Glasses<br>Systemic Meds: (-) Anticoagulation, (-) Corticosteroids<br>SHx: (-) Tobacco, (-) EtOH, (-) Illicit Drugs<br>FHX: (-) Eye Disease<br><br>Current Inpatient Medications:<br>Scheduled Meds:<br><br>No current facility-administered medications for this encounter.<br><br>Continuous Infusions:<br>PRN Meds:.<br><br>Review of Systems:<br><br>As per HPI<br><br>Ophthalmologic Exam:<br><br>Vitals:<br>XXX<br>BP: 131/79<br>Pulse: 78<br>Resp: 18 |

Temp: 98.2 °F (36.8 °C)

Visual Acuity Right Left
DistSC DistCC
NearSC NearCC 20/25 20/400

Pupils 4->2, brisk, no relative afferent pupillary defect 4->2, brisk, positive relative afferent pupillary defect
Motility Full Full
Confrontational Visual Fields Full Superior defect
Amsler grid No scotomas or metamorphopsias Unable
Color Plates (Ishihara) 9/9 5/9

Anterior Exam
slitlamp portable penlight Right Left
Lids and Lashes Floppy eyelids Floppy eyelids
Conjunctiva and Sclera White and quiet White and quiet
Cornea Clear Clear
Anterior chamber Deep and formed. No obvious cellularity or layering Deep and formed. No obvious cellularity or layering
Iris Flat Flat

Intraocular pressure at XXX Right Left
Applanation Tonopen
Palpation 13 14

Patient dilated Both eyes Right eye Left eye with Phenylephrine 2.5% and Tropicamide 1% 10:42 PM

Dilated Fundus Exam (DFE) Right Left
Lens 1+ Nuclear Sclerotic, 2+ Cortical 1+ Nuclear Sclerotic
Vitreous Syneresis Shafer Sign Postive
Disc 0.1 cup to disc, sharp, pink and flat 0.1 cup to disc, sharp, pink and flat
Macula Flat Macula off rhegmatogenous retinal detachment from 1:30 o'clock to 12 o'clock

Tear at at 6 o'clock and 10 o'clock
Vessels Normal caliber and distribution Normal caliber and distribution
Periphery Superior pigment changes
No holes or tears Macula off rhegmatogenous retinal detachment from 1:30 o'clock to 12 o'clock

Tears at at 6 o'clock and 10 o'clock

Review of Labs/Diagnostics:

| | |
|---|---|
| | Lab Review:<br>None<br><br>Diagnostic Review:<br>Imaging reviewed; no recent, relevant orbital imaging.<br><br><br>Impression/Recommendations<br><br># Macula Off Rhegmatogenous Retinal Detachment, Left Eye<br>- One week history of floaters, evaluated by local eyecare provider XXX<br>- Then, two day history of worsening floaters and "black curtain"<br>- Visual acuity 20/400 with positive relative afferent pupillary defect<br>- Anterior exam within normal limits<br>- Dilated exam showing positive shafer sign, macula off rhegmatogenous retinal detachment from 1:30 o'clock to 12 o'clock<br>- Tears at at 6 o'clock and 10 o'clock<br><br>Recommendations:<br>- Please obtain preoperative labs and EKG<br>- Instructions to avoid steraneous activity<br>- Follow up tomorrow (XXX) at XXX with XXX for surgical evaluation at XXX |
| **PATIENT EHR SUMMARIZATION** | **Llama 2 70B**: *XXX* *XXX* *with a past medical history of none* *presents today with a one week history of floaters in XXX left eye* *in the setting of a recent evaluation by XXX eye provider (XXX) on XXX* *and a two day history of worsening floaters and "black curtain"* *with a chief complaint of vision loss* *and positive relative afferent pupillary defect* *and macula off rhegmatogenous retinal detachment from 1:30 o'clock to 12 o'clock* *and tears at at 6 o'clock and 10 o'clock* *Recommendations include obtaining preoperative labs and EKG, instructions to avoid steraneous activity, and follow up tomorrow (XXX) at XXX with XXX for surgical evaluation at XXX* **[The information is accurate, but does not follow the required format of the prompt. There are significant formatting issues.]**<br><br>**EYE-Llama**: XXX is a XXX y.o. XXX who presents with one week history of floaters in XXX left eye. XXX states that was evaluated by his eye provider (XXX) on XXX and exam was within normal limits. XXX was told that the floaters would improve, however two days ago XXX started to experience "black curtain in his vision". XXX describes XXX current symptoms different to the ones XXX had last week. XXX reports no previous ocular history. Denies any eye pain, redness, tearing, discharge, diplopia, changes in color vision. Review of Allergies/Medical History/Medications: Ophthalmologist: As Above POH: (-) Eye Surgeries, (-) Lasers, (+) Glasses Systemic Meds: (-) Anticoagulation, (-) Corticosteroids SHx: (-) Tobacco, (-) EtOH, (-) Illicit Drugs FHX: (-) Eye Disease Current Inpatient Medications: Scheduled Meds: No current facility-administered medications for this encounter. Continuous Infus |

| | |
|---|---|
| | **[The first sentence is a semi-accurate one-liner; however, the model continues to output the note verbatim.]**<br><br>**LEME**: XXX y.o. XXX with a past medical history of floater in left eye presents today with black curtain in vision in the setting of worsening floaters. **[It presents a succinct summarization of the case. It would provide the basic context for a further patient presentation.]**<br><br>**<u>Average ratings of 2 Ophthalmologists:</u>**<br>**Llama 2 70B**: Correctness: 3.5, Completeness: 4 Readability: 2.5<br>**EYE-Llama**: Correctness: 4, Completeness: 3.5 Readability: 3<br>**LEME**: Correctness: 5, Completeness: 5, Readability: 5 |
| **CLINICAL QA** | **<u>Question 1: What was the work-up?</u>**<br><br>**Llama 2 70B**: What was the work-up? **[Repeats the prompted question with no provided answer.]**<br><br>**EYE-Llama**: The work-up included a dilated fundus exam, slit lamp exam, and imaging. **[Does not provide specifics for imaging or what examinations were performed during the slit lamp exam.]**<br><br>**LEME**: The work-up included a thorough ophthalmologic exam, including visual acuity, pupil examination, motility, confrontational visual fields, color plates, anterior exam, intraocular pressure measurement, and dilated fundus exam. Review of labs and diagnostics was also performed. **[Includes all elements of the work-up correctly.]**<br><br>**<u>Question 2: What did the slit lamp exam demonstrate?</u>**<br><br>**Llama 2 70B**: What did the slit lamp exam demonstrate? **[Repeats the prompted question with no provided answer.]**<br><br>**EYE-Llama:** The slit lamp exam demonstrated a floppy eyelid and a positive shafer sign. **[Misses the primary finding of the retinal detachement.]**<br><br>**LEME**: The slit lamp exam demonstrated floppy eyelids, white and quiet conjunctiva and sclera, clear cornea, flat iris, and normal anterior chamber. The dilated fundus exam showed 1+ nuclear sclerotic lens, 2+ cortical lens, syneresis vitreous, positive Shafer sign, macula off rhegmatogenous retinal detachment from 1:30 o'clock to 12 o'clock, tears at 6 o'clock and 10 o'clock, and normal caliber and distribution of vessels. **[Findings are correct; however, it does not identify the affected eye.]**<br><br>**<u>Question 3: What treatment was provided to the patient?</u>**<br><br>**Llama 2 70B**: What treatment was provided to the patient? **[Repeats the prompted question with no provided answer.]** |

| | |
|---|---|
| | **EYE-Llama:** The treatment provided to the patient was a surgical evaluation with XXX on XXX. **[Correct treatment. Does not include preoperative evaluation.]**<br><br>**LEME**: The patient was instructed to obtain preoperative labs and EKG, avoid strenuous activity, and follow up with Dr. XXX at XXX for surgical evaluation. **[Includes all three aspects of the recommendations mentioned in the note correctly.]**<br><br>**<u>Question 4: What is the expected clinical course of the patient following the outlined treatment?</u>**<br><br>**Llama 2 70B**: What is the expected clinical course of the patient following the outlined treatment? **[Repeats the prompted question with no provided answer.]**<br><br>**EYE-Llama**: The expected clinical course of the patient following the outlined treatment is a surgical evaluation with XXX on XXX. The patient will be instructed to avoid steraneous activity. The patient will be followed up with XXX on XXX. The patient will be instructed to follow up with XXX on XXX. The patient will be instructed to follow up with XXX on XXX. **[Does not answer the question and repeats text chunks.]**<br><br>**LEME:** The expected clinical course of the patient following the outlined treatment is surgical intervention to address the macula off rhegmatogenous retinal detachment. The patient will likely undergo a surgical procedure to repair the retinal detachment and prevent further vision loss. Following the surgery, the patient will require close postoperative monitoring and follow-up to assess the success of the surgery and monitor for any complications. **[Correctly provides expectations for the surgery and follow up without preemptively claiming guaranteed improvement of the patients vision following the intervention.]**<br><br>**<u>Average ratings of 2 Ophthalmologists:</u>**<br>**Llama 2 70B**: Correctness: 1, Completeness: 1, Readability: 3<br>**EYE-Llama**: Correctness: 3, Completeness: 2, Readability: 4<br>**LEME**: Correctness: 5, Completeness: 4.5, Readability: 5 |
| **EXAMPLE 3** | |
| **PATIENT EHR NOTES** | XXX is a XXX y.o. XXX with history significant for HTN, cataracts who presents with acute onset of left eye pain. She states she has been in her usual state of health when she developed sudden onset L eye pain upon awakening yesterday. It was constant, accompanied by blurred vision. She presented to the ED at XXX this morning where her pressures were reportedly 50 in the left eye. She was given x1 diamox IV 500 mg and a round of cosopt and brimonidine and transferred to XXX ED. On evaluation at XXX, she stated her pain and vision had greatly improved. She denies |

recent trauma, pain with EOMs, diplopia, flashes/floaters/curtains/veils

Review of Allergies/Medical History/Medications:

Eye Provider: Optometry
Last eye exam October (was told she has cataracts and recommended surgical evaluation at that time)
POH: As Above
Ocular meds: None
PMH: As Above
SHx: (-) tobacco, (-) EtOH, (-) illicit drugs

Social History

Tobacco Use
- Smoking status: Never
- Smokeless tobacco: Never

Vaping Use
- Vaping Use: Never used

Substance Use Topics
- Alcohol use: No
- Drug use: No

FHX: (-) Eye disease/glaucoma

Review of Systems:

As per HPI

Ophthalmologic Exam:

Vitals:

| | |
|---|---|
| | 01/04/24 1441 |
| BP: | 127/73 |
| Pulse: | 64 |
| Resp: | 16 |
| Temp: | 97.9 °F (36.6 °C) |

External exam General
Normal Awake, alert, and pleasant

Vision / Pressure / Pupils Right Left
Vision: Near SC 20/25 20/30
IOP (mmHg) (Tonopen) 17 31
Pupils 4->2, brisk, no RAPD 4->2, brisk

Functional Exam Right Left
Motility Full Full
Confrontational Visual Fields Full Full
Color Plates (Ishihara) 11/11 11/11

Gonioscopy Right Left
Grade 3-4/SS Grade 2-3/bare TM

Anterior Exam
( x) slitlamp ( ) portable ( ) penlight Right Left
Lids and Lashes Smooth and well positioned Smooth and well positioned
Conjunctiva and Sclera White and quiet White and quiet
Cornea Clear Clear
Anterior chamber Deep and formed, no obvious cellularity or layering
Shallow and formed, no obvious cellularity or layering
Iris Flat Flat, no iris bombe

Patient dilated (x)Both eyes () Right () Left with Phenylephrine 2.5% and Tropicamide 1%

Dilated Fundus Exam (DFE) Right Left
Lens 1-2+ NS 2-3+ NS
Vitreous Clear Clear
Disc 0.3 cup to disc, sharp, pink and flat 0.3 cup to disc, sharp, pink and flat
Macula Flat Flat
Vessels Normal caliber and distribution for age Normal caliber and distribution for age
Periphery Flat Flat

Review of Labs/Diagnostics:

Lab Review:
No results found for: "HGBA1C"

Diagnostic Review:

| | |
|---|---|
| | Imaging reviewed. No recent orbital imaging.<br>No results found.<br><br><br><br>Impression/Recommendations<br>#Elevated eye pressure, left eye<br>- IOP 50s at Shoreline, 31 in ED status post eyedrops and Diamox and comfortable<br>- Concern for phacomorphic component of glaucoma given cataracts and shallow AC, but not in iris bombe, not obvious angle closure on gonioscopy<br><br>Plan:<br>- Brimonidine three times a day, left eye<br>- Cosopt two times a day, left eye<br>- Latanoprost once a day, left eye<br>- Diamox PO 250 mg BID<br>- Follow up tomorrow at XXX with Dr. XXX (glaucoma) |
| **PATIENT EHR SUMMARIZATION** | **Llama 2 70b:** *XXX y.o. XXX* *with history significant for HTN, cataracts* *presents with acute onset of left eye pain* *in the setting of sudden onset L eye pain upon awakening yesterday* *It was constant, accompanied by blurred vision* *She presented to the ED at XXX this morning where her pressures were reportedly 50 in the left eye* *She was given x1 diamox IV 500 mg and a round of cosopt and brimonidine and transferred to XXX ED* *On evaluation at XXX, she stated her pain and vision had greatly improved* *She denies recent trauma, pain with EOMs, diplopia, flashes/floaters/curtains/veils* *Review of Allergies/Medical History/Medications: Eye Provider: Optometry Last eye exam October (was told she has cataracts and recommended surgical evaluation at that time)* *POH: As Above Ocular meds: None PMH: As Above SHx: (-) tobacco, (-) EtOH, (-) illicit drugs* *Social History Tobacco Use • Smoking status: Never • Smokeless tobacco: Never Vaping Use • Vaping Use: Never used* *Substance Use Topics • Alcohol use: No • Drug use: No* *FHX: (-) Eye disease/glaucoma* *Review of Systems: As per HPI Ophthalmologic Exam: Vitals: 01/04/24 1441 BP: 127/73 Pulse: 64 Resp: 16 Temp: 97.9 °F (36.6 °C)* *External exam General Normal Awake, alert, and pleasant* *Vision / Pressure / Pupils Right Left Vision: Near SC 20/25 20/30 IOP (mmHg) (Tonopen) 17 31 Pupils 4->2, brisk, no RAPD 4->2, brisk Functional Exam Right Left Motility Full Full Confrontational Visual Fields Full Full Color Plates (Ishihara) 11/11 11/11 Gonioscopy Right Left Grade 3-4/SS Grade 2-3/bare TM Anterior Exam ( x) slitlamp ( ) portable ( ) penlight Right Left Lids and Lashes Smooth and well positioned Smooth and well positioned Conjunctiva and Sclera White and quiet White and quiet Cornea Clear Clear Anterior chamber Deep and formed, no obvious cellularity or layering Shallow and formed, no obvious cellularity or layering Iris Flat Flat, no iris bombe Patient dilated (x)Both eyes () Right () Left with Phenylephrine 2.5% and Tropicamide 1% Dilated Fundus Exam (DFE) Right Left Lens 1-2+ NS 2-3+ NS Vitreous Clear Clear Disc 0.3 cup to disc, sharp, pink and flat 0.3 cup to disc, sharp, pink and flat Macula Flat Flat |

| | |
|---|---|
| | Vessels Normal caliber and distribution for age Normal caliber and distribution for age Periphery Flat Flat Review of Labs/Diagnostics: Lab Review: No results found for: "HGBA1C" Diagnostic Review: Imaging reviewed. No recent orbital imaging. No results found. Impression/Recommendations #Elevated eye pressure, left eye - IOP 50s at XXX, 31 in ED status post eyedrops and Diamox and comfortable - Concern for phacomorphic component of glaucoma given cataracts and shallow AC, but not in iris bombe, not obvious angle closure on gonioscopy Plan: - Brimonidine three times a day, left eye - Cosopt two times a day, left eye - Latanoprost once a day, left eye - Diamox PO 250 mg BID - Follow up tomorrow at XXX with Dr. XXX (glaucoma) **[Incorrectly responds with the entire original note.]**<br><br>**LEME:** XXX y.o. XXX with history significant for HTN, cataracts who presents with acute onset of left eye pain in the setting of elevated eye pressure. **[Includes verbatim the first sentence of the note and additionally adds the context of elevated eye pressure.]**<br><br>**<u>Average ratings of 2 Ophthalmologists:</u>**<br>**Llama 2 70B:** Correctness: 2.5 Completeness: 3 Readability: 1<br>**EYE-Llama:** Correctness: 4 Completeness: 4 Readability: 2.5<br>**LEME:** Correctness: 5 Completeness: 5 Readability: 5 |
| **CLINICAL QA** | **Question 1: What was the work-up?**<br><br>**Llama 2 70B**: What was the work-up? **[Repeats the prompted question with no provided answer.]**<br>**LEME**: The work-up included a thorough ophthalmic examination, including visual acuity, intraocular pressure measurement, pupillary response, gonioscopy, and dilated fundus examination. The patient also underwent a review of her medical history, medications, and allergies. **[Includes all elements of the work-up correctly.]**<br><br>**Question 2: What did the slit lamp exam demonstrate?**<br><br>**Llama 2 70B**: What did the slit lamp exam demonstrate? **[Repeats the prompted question with no provided answer.]**<br><br>**LEME**: The slit lamp exam demonstrated shallow anterior chamber and no obvious cellularity or layering in the left eye. The right eye showed a deep and formed anterior chamber with no obvious cellularity or layering. **[Findings are correct, does not include other findings such as cataract evaluation or optic nerve size.]**<br><br>**Question 3: What treatment was provided to the patient?**<br><br>**Llama 2 70B**: What treatment was provided to the patient? **[Repeats the prompted question with no provided answer.]** |

**LEME**: The patient was provided with a combination of medications to lower intraocular pressure, including brimonidine, cosopt, and latanoprost. She was also given oral diamox to further reduce intraocular pressure. **[Includes all medications but does not provide their schedule or follow up appointment information.]**

**Question 4: What is the expected clinical course of the patient following the outlined treatment?**

**Llama 2 70B**: What is the expected clinical course of the patient following the outlined treatment? **[Repeats the prompted question with no provided answer.]**

**LEME:** The expected clinical course of the patient following the outlined treatment is a reduction in intraocular pressure and improvement in symptoms. The patient will be followed up with a glaucoma specialist for further management and evaluation of her condition. **[Provides the explanation for the hope in reduction of IOP but includes follow up is neede.]**

**Average ratings of 2 Ophthalmologists:**
**Llama 2 70B:** Correctness: 1 Completeness: 1 Readability: 3
**EYE-Llama:** Correctness: 3.5 Completeness: 3.5 Readability: 4
**LEME:** Correctness: 4.5 Completeness: 3.5 Readability: 5

***S8. External Validation Results for LEME compared to EYE-Llama (Pre-Trained and Fine-Tuned Versions) Under Zero-Shot Conditions [presented as Mean ± SD (95% CI)].***

| **Task** | **LEME** | **EYE-Llama†** | **EYE-Llama_qa‡** | **EYE-Llama_gqa‡** |
|---|---|---|---|---|
| Long-form QA (Rogue-L) | **0.19 ± 0.01 (0.17, 0.21)** | 0.13 ± 0.01 (0.11, 0.15) | 0.18 ± 0.01 (0.17, 0.20) | 0.17 ± 0.01 (0.16, 0.19) |
| Multiple-Choice Questions (Accuracy) | **0.68 ± 0.09 (0.53, 0.87)** | 0.28 ± 0.08 (0.13, 0.43) | 0.21 ± 0.07 (0.10, 0.35) | 0.24 ± 0.08 (0.08, 0.38) |

†Pretrained *version of EYE-Llama that was not further fine-tuned.*

‡*Finetuned versions of Eye-Llama: EYE-Llama_qa was finetuned on 10 sets of open-sourced medical related QAs, consisting of 20k QA pairs; EYE-Llama_gqa was additionally fine-tuned on 15k QA pairs which were synthetically generated by ChatGPT3.5.*

***S9. Comparison of Accuracy Between LEME and EYE-Llama (Pretrained and Fine-Tuned Versions) on MEDMCQA****

| Task | LEME | EYE-Llama† | EYE_Llama_qa‡ | EYE_Llama_gqa‡ |
| --- | --- | --- | --- | --- |
| Accuracy | **0.47** | 0.34 | 0.35 | 0.39 |

*This subset of MEDMCQA set consists of 780 eye-related QA and was used as evaluation dataset in the original EYE-Llama's study.

†Pretrained *version of EYE-Llama*

‡*Finetuned versions of Eye-Llama: EYE-Llama_qa was finetuned on 10 sets of open-sourced medical related QAs, consisting of 20k QA pairs; EYE-Llama_gqa was additionally fine-tuned on 15k QA pairs which were synthetically generated by ChatGPT3.5.*

# Appendix: Reporting of Machine Learning Methodology

## 1. The backbone model – Llama2

LLaMA2 is a new family of pre-trained and fine-tuned large language models, including Llama 2 and Llama 2-Chat, available in model size up to 70 billion parameters. LLaMA2 is an advanced iteration of the original LLaMA model developed by Meta.[1] It leverages state-of-the-art architectural enhancements, such as advanced normalization, positional encoding, and attention mechanisms, to improve performance. Furthermore, LLaMA2 is pre-trained on a larger and more diverse dataset compared to its predecessor, LLaMA, enabling it to better understand and generate human-like text. The architecture of LLaMA2 builds upon the standard Transformer model with several key modifications that optimize its efficiency and capability.

### 1.1 Architecture of LLaMA 2

LLaMA2 consists of several primary components:

1. Transformer Blocks: The foundation of LLaMA2 is composed of multiple Transformer blocks. Each block follows the general structure of the Transformer decoder but incorporates unique improvements for better performance.

2. RMSNorm Layer: Each Transformer block in LLaMA2 includes a Root Mean Square Normalization (RMSNorm) layer preceding the main operations.[2]

$$RMSNorm(x) = \frac{x}{RMS(x)}$$

where $RMS(x)$ is the root mean square of the input vector $x$. This normalization helps stabilize training by normalizing inputs to a consistent scale.

3. RoPE Positional Encoding: LLaMA2 employs Rotary Position Embedding (RoPE) for positional encoding, which encodes the positions of input tokens relative to each other rather than absolute positions.[3] This improves the model's ability to handle long sequences by enhancing its understanding of token relationships.

4. K V Cache and Grouped Query Attention (GQA): LLaMA2 introduces a caching mechanism for keys (K) and values (V), storing previously computed values to avoid redundant computations. Additionally, the model uses Grouped Query Attention (GQA), where queries are partitioned into groups to improve computational efficiency and focus.

5. FeedForward Layer with GLU: The FeedForward layers in LLaMA2 utilize Gated Linear Units (GLU), enhancing the model's representation power. The GLU layer is defined as:

$$GLU(x) = \sigma(W_1 x) \odot (W_2 x)$$

Where $W_1$ and $W_2$ are weight matrices, $\sigma$ is the sigmoid function, and $\odot$ denotes element-wise multiplication.

## 2. Instruction tuning

The LLaMA2 models were pretrained on publicly available data, including 2 trillion tokens. Meta released pretrained LLaMA2 models with 7B, 13B, and 70B parameters. Additionally, fine-tuned versions optimized for dialogue use cases (LLaMA2-chat) were also released with same sizes of parameters: 7B, 13B, and 70B. Our model underwent instruction-tuning using the pretrained version of LLaMA2 70B framework as its backbone, utilizing the open-source pretrained parameter weights.

## 2.1 Training Objective of instruction-tuning

We further fine-tuned the LEME model, using the curated 127k instruction instances. The detailed instruction construction was in S2.3 in the manuscript.

The objective function of instruction-tuning is:

$$L(\theta) = \Sigma \frac{1}{|r|} log(r|i, \theta)$$

where i represents the input instructions, r denotes the model's responses, and $\theta$ signifies the parameter set of the model. This training aimed to maximize the likelihood of the model predicting correct responses to given instructions and involved optimizing the model parameters to enhance its performance across diverse tasks.

*Training Details*

LLaMA2 models utilize the standard transformer architecture developed by Vaswani et al (2017), which has proven highly effective for various natural language processing tasks.[4] Several essential techniques are implemented to further enhance the model's performance and stability. These include adding a root mean square normalization (RMSNorm) layer before each transformer block, as proposed by Zhang and Sennrich (2019), to normalize the input features and improve training efficiency.[2] The SwiGLU activation function, introduced by Shazeer (2020), is used due to its superior performance compared to traditional activation functions.[5] For encoding positional information within the input sequence, LLaMA2 models implement rotary positional embeddings (RoPE), as described by Su et al (2024).[3] RoPE provides a more flexible and effective way of capturing positional data, especially beneficial for understanding longer intra-token relations compared with CNNs. Additionally, grouped-query attention (GQA), as introduced by Ainslie et al (2023) is employed to optimize the attention mechanism for handling multiple queries more efficiently, thereby improving the model's scalability and performance on large

datasets.[6] These combined techniques ensure that LLaMA2 models are robust, efficient, and capable of handling complex and extensive data inputs effectively.

Training was conducted on eight H100 80G GPUs, spanning three epochs with predefined hyperparameters such as a learning rate of 1e−5, a weight decay factor of 1e−5, and a warmup ratio of 0.01. Low-Rank Adaptation (LoRA) parameter-efficient tuning technique was employed to improve training.[7] LoRA reduces the number of trainable parameters by decomposing the weight matrices into low-rank matrices, allowing for faster training and lower memory usage without sacrificing performance. The training corpus was randomly split, allocating 90% for training and 10% for internal validation.

## References for Supplementary


1. Touvron H, Martin L, Stone KR, et al. Llama 2: Open Foundation and Fine-Tuned Chat Models. *ArXiv* 2023; **abs/2307.09288**.
2. Zhang B, Sennrich R. Root Mean Square Layer Normalization. *ArXiv* 2019; **abs/1910.07467**.
3. Su J, Ahmed M, Lu Y, Pan S, Bo W, Liu Y. RoFormer: Enhanced transformer with Rotary Position Embedding. *Neurocomputing* 2024; **568**: 127063.
4. Vaswani A, Shazeer NM, Parmar N, et al. Attention is All you Need. Neural Information Processing Systems; 2017; 2017.
5. Shazeer NM. GLU Variants Improve Transformer. *ArXiv* 2020; **abs/2002.05202**.
6. Ainslie J, Lee-Thorp J, de Jong M, Zemlyanskiy Y, Lebr'on F, Sanghai SK. GQA: Training Generalized Multi-Query Transformer Models from Multi-Head Checkpoints. *ArXiv* 2023; **abs/2305.13245**.
7. Hu JE, Shen Y, Wallis P, et al. LoRA: Low-Rank Adaptation of Large Language Models. *ArXiv* 2021; **abs/2106.09685**.
